# Revisão Sistemática: Antagonistas dos Receptores de Mineralocorticoides na Insuficiência Cardíaca com Fração de Ejeção Preservada e Levemente Reduzida

**DOI:** 10.36660/abc.20250622

**Published:** 2026-05-26

**Authors:** Marcus Vinicius Simões, Luiz Claudio Danzmann, Sílvia Marinho Martins, José Albuquerque de Figueiredo, Fabiana G. Marcondes-Braga, Ricardo Mourilhe-Rocha, Denilson Campos de Albuquerque, Mucio Tavares de Oliveira, Lidia Ana Zytynski Moura

**Affiliations:** 1 Universidade de São Paulo Faculdade de Medicina de Ribeirão Preto Ribeirão Preto SP Brasil Universidade de São Paulo Faculdade de Medicina de Ribeirão Preto, Ribeirão Preto, SP – Brasil; 2 Universidade Luterana do Brasil Canoas RS Brasil Universidade Luterana do Brasil, Canoas, RS – Brasil; 3 Hospital São Lucas da PUCRS Porto Alegre RS Brasil Hospital São Lucas da PUCRS, Porto Alegre, RS – Brasil; 4 Pronto Socorro Cardiológico de Pernambuco Recife PE Brasil Pronto Socorro Cardiológico de Pernambuco (PROCAPE), Recife, PE – Brasil; 5 Real Hospital Português de Beneficência em Pernambuco Recife PE Brasil Real Hospital Português de Beneficência em Pernambuco – Realcor, Recife, PE – Brasil; 6 Universidade Federal do Maranhão São Luís MA Brasil Universidade Federal do Maranhão, São Luís, MA – Brasil; 7 Instituto do Coração do Hospital das Clínicas Faculdade de Medicina Universidade de São Paulo São Paulo SP Brasil Instituto do Coração do Hospital das Clínicas da Faculdade de Medicina da Universidade de São Paulo, São Paulo, SP – Brasil; 8 Universidade do Estado do Rio de Janeiro Rio de Janeiro RJ Brasil Universidade do Estado do Rio de Janeiro, Rio de Janeiro, RJ – Brasil; 9 Hospital Pró-Cardíaco – Américas Serviços Médicos Rio de Janeiro RJ Brasil Hospital Pró-Cardíaco – Américas Serviços Médicos, Rio de Janeiro, RJ – Brasil; 10 Pontifícia Universidade Católica do Paraná Curitiba PR Brasil Pontifícia Universidade Católica do Paraná, Curitiba, PR – Brasil

**Keywords:** Antagonistas de Receptores de Mineralocorticoides, Insuficiência Cardíaca Diastólica, Insuficiência Cardíaca Sistólica, Revisão Sistemática, Metanálise

## Abstract

A insuficiência cardíaca com fração de ejeção preservada (ICFEp) ou levemente reduzida (ICFElr) é uma condição prevalente associada à significativa morbidade e mortalidade em que os benefícios dos antagonistas dos receptores de mineralocorticoides (ARMs) ainda não são claros. Nosso objetivo foi desenvolver uma revisão sistemática sobre o uso de ARMs em pacientes com ICFEp ou ICFElr. Foi realizada uma revisão sistemática com metanálise, utilizando uma estratégia de busca ampla nas bases de dados MEDLINE, Embase e Cochrane Central Register of Controlled Trials (CENTRAL). Foram incluídos ensaios clínicos randomizados que avaliaram o uso de ARMs em pacientes com ICFEp, comparados a grupos controle. A qualidade da evidência e a força da recomendação foram avaliadas segundo a metodologia Grading of Recommendations Assessment, Development, and Evaluation (GRADE). Oito estudos foram incluídos. Não foram observadas diferenças estatisticamente significativas para os desfechos de mortalidade geral e mortalidade cardiovascular em comparação ao grupo controle para qualquer dos ARMs. Identificou-se benefício na redução do risco de hospitalização por insuficiência cardíaca (risco relativo: 0,87; intervalo de confiança de 95%: 0,79 a 0,96; certeza da evidência moderada) e piora da insuficiência cardíaca (risco relativo: 0,83; intervalo de confiança de 95%: 0,77 a 0,89; certeza da evidência alta). Os resultados da metanálise indicam significativo benefício dos ARMs, espironolactona ou finerenona, para redução de hospitalizações e eventos de piora da insuficiência cardíaca em pacientes com ICFEp ou ICFElr.

## Graphical abstract

**Figura Central:**
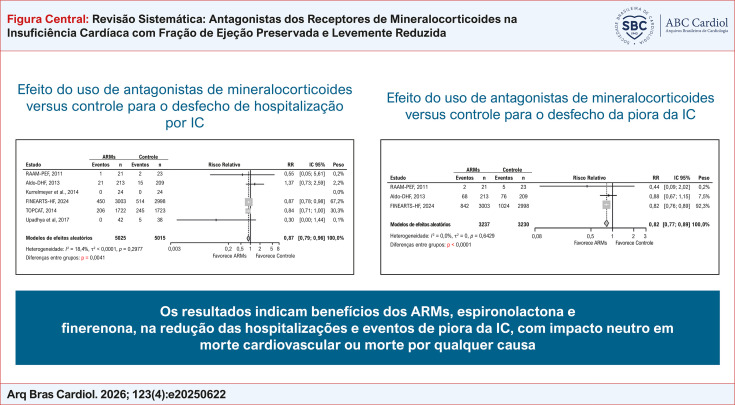
: Revisão Sistemática: Antagonistas dos Receptores de Mineralocorticoides na Insuficiência Cardíaca com Fração de Ejeção Preservada e Levemente Reduzida FEVE: fração de ejeção do ventrículo esquerdo; ICFElr: insuficiência cardíaca com fração de ejeção levemente reduzida; ICFEp: insuficiência cardíaca com fração de ejeção preservada; TFGe: taxa de filtração glomerular estimada.

## Introdução

Em pacientes com insuficiência cardíaca com fração de ejeção reduzida, o uso de antagonistas esteroidais dos receptores mineralocorticoides (ARMs), nominalmente espironolactona e eplerenona, tem demonstração clara da redução do risco de morte e hospitalização. Evidência desse efeito é derivada de dois estudos clínicos fundamentais: o Randomized Aldactone Evaluation Study (RALES)^1^ e o Eplerenone in Mild Patients Hospitalization and Survival Study in Heart Failure (EMPHASIS-HF).^2^ Dessa forma, as diretrizes internacionais trazem recomendações fortes e consistentes para o uso desses ARMs em pacientes com insuficiência cardíaca com fração de ejeção reduzida.^3^

Em contrapartida, a eficácia desses agentes para tratamento da insuficiência cardíaca com fração de ejeção levemente reduzida (ICFElr) ou preservada (ICFEp) ainda é incerta. Até recentemente, um único estudo clínico randomizado multicêntrico, o estudo Treatment of Preserved Cardiac Function Heart Failure with an Aldosterone Antagonist (TOPCAT),^4^ havia testado o efeito da espironolactona em pacientes com fração de ejeção do ventrículo esquerdo (FEVE) > 45%. Neste estudo, a espironolactona não reduziu significativamente o desfecho primário composto por primeira hospitalização por insuficiência cardíaca, parada cardíaca ressuscitada ou morte cardiovascular. Contudo, a análise dos componentes do desfecho primário mostrou redução nas hospitalizações por insuficiência cardíaca, indicando potencial efeito significativo da espironolactona.^4^ Adicionalmente, análises posteriores levantaram aspectos que poderiam justificar um resultado neutro deste estudo, observando-se que uma parcela considerável dos pacientes incluídos nesse estudo nos centros da Rússia e Geórgia talvez não tivesse insuficiência cardíaca, já que a taxa de eventos observados foi bem menores que o observado nos pacientes incluídos nos 4 países das Américas (Estados Unidos, Canadá, Brasil e Argentina).^5^ Outra análise post hoc indicou que muitos pacientes não devem ter tomado a medicação atribuída, tendo-se em muitos pacientes randomizados para espironolactona não se detectado metabólito da droga na urina, indicando um possível benefício naqueles que efetivamente a utilizaram.^6^ Como consequências desses aspectos controversos, as recomendações para uso de ARMs na ICFElr e ICFEp são fracas ou inexistentes.^7^

Recentemente, o estudo Finerenone Trial to Investigate Efficacy and Safety Superior to Placebo in Patients With Heart Failure (FINEARTS-HF) avaliou a eficácia da finerenona, um ARM não esteroidal, em pacientes com FEVE > 40%, mostrando redução significativa do risco do desfecho primário composto por eventos totais de piora da insuficiência cardíaca e morte cardiovascular em pacientes com ICFEp e ICFElr.^8^

Portanto, diante da crescente evidência sugerindo potenciais benefícios dos ARMs em pacientes com insuficiência cardíaca de fração de ejeção não reduzida, foi realizada uma revisão sistemática com o objetivo de identificar, avaliar e sumarizar as evidências científicas disponíveis quanto à eficácia e segurança desses fármacos em pacientes com ICFEp e ICFElr.

## Métodos

Para a elaboração desta revisão sistemática da Sociedade Brasileira de Cardiologia (SBC), foi realizada uma revisão sistemática rápida, seguindo os critérios da Cochrane.^9^ O protocolo da revisão foi registrado na plataforma Open Science Framework, sob o número 10.17605/OSF.IO/MVB75. O relato do estudo esteve de acordo com as diretrizes do Preferred Reporting Items for Systematic Reviews and Meta-Analyses (PRISMA).^10^

A questão de pesquisa, estruturada no formato PICO, buscou avaliar a eficácia e segurança do uso de ARMs em pacientes com ICFEp e ICFElr quando comparado ao tratamento usual ou terapia otimizada.

Os critérios de inclusão foram: (1) ensaios clínicos randomizados (ECRs); (2) pacientes diagnosticados com ICFEp (FEVE ≥ 40%); (3) pacientes sintomáticos classificados na classe funcional II, III ou IV da New York Heart Association; (4) estudos que avaliem o uso de ARMs como intervenção e incluam grupo controle utilizando placebo ou tratamento padrão para ICFEp.

Os critérios de exclusão aplicados foram: (1) pacientes com taxa de filtração glomerular estimada < 25 mL/min/1,73 m^2^; (2) pacientes com níveis séricos de potássio > 5,0 mmol/L; (3) estudos que não apresentem um grupo comparador adequado (placebo ou tratamento padrão); (4) revisões narrativas, relatos de caso, editoriais e estudos pré-clínicos (em animais ou in vitro).

Os desfechos primários de eficácia incluíram mortalidade geral, mortalidade cardiovascular, hospitalização por insuficiência cardíaca, piora da insuficiência cardíaca e qualidade de vida. Os desfechos de segurança analisados foram hipercalemia e deterioração da função renal.

Inicialmente, foi realizada uma busca por revisões sistemáticas sobre o tema em 3 bases de dados: Embase, MEDLINE (via PubMed) e Cochrane Library na data de 26 de fevereiro de 2025. Detalhes sobre a metodologia e as estratégias de busca estão disponíveis no material suplementar (Quadro 1S). Contudo, devido à ausência de revisões sistemáticas de alta qualidade metodológica para embasar as recomendações, uma nova busca por ECRs foi realizada nas mesmas bases de dados, incluindo também uma busca manual complementar, baseada nas revisões sistemáticas selecionadas na primeira etapa. O material suplementar (Quadro 2S e a Figura S1) apresentam detalhes sobre a busca por revisões sistemáticas.

A busca por ECR foi restrita a estudos publicados em inglês e português. Detalhes sobre a estratégia de busca estão descritos no material suplementar (Quadro 3S). A seleção dos estudos foi realizada utilizando o gerenciador de referências Rayyan^11^ e avaliada por um único autor. A triagem dos textos completos também foi realizada pelo mesmo revisor. A extração de dados foi conduzida por um revisor utilizando um formulário previamente testado e validada por um segundo revisor.

### Análise estatística

Os dados foram sintetizados de forma narrativa; quando possível, foi conduzida uma metanálise para cada desfecho de interesse, sumarizando os resultados obtidos nos ECRs.

As metanálises foram realizadas utilizando um modelo de efeitos randômicos, com dados dos ensaios clínicos incluídos. Para desfechos dicotômicos, foi utilizado o risco relativo (RR) como medida de estimativa do tamanho do efeito. Para desfechos contínuos, foram utilizadas a diferença de médias (DM). Um intervalo de confiança de 95% foi considerado. A heterogeneidade foi avaliada pelo valor de I^2^, considerando valores acima de 50% indicativos de heterogeneidade significativa. Metanálises foram conduzidas no software estatístico R, versão 4.4.3, utilizando o pacote meta, versão 8.0-2.^12^

Foram planejadas análises de subgrupos para os desfechos primários, desde que houvesse disponibilidade de dados nos estudos incluídos. Também estavam previstas análises de sensibilidade para os desfechos primários, a serem realizadas caso houvesse um número suficiente de estudos metodologicamente homogêneos, com exclusão daqueles que apresentassem ao menos um domínio avaliado como de alto risco ou risco incerto de viés. No entanto, tais análises não foram necessárias, uma vez que os estudos incluídos apresentaram baixa heterogeneidade metodológica e de resultados. A avaliação do viés de publicação teria sido realizada por meio da inspeção visual dos gráficos em funil, caso pelo menos 10 estudos fossem agrupados em uma metanálise.

### Avaliação do risco de viés e certeza da evidência

As potenciais revisões sistemáticas foram avaliadas quanto a sua qualidade metodológica através da ferramenta AMSTAR-2.^13^ O risco de viés dos ECRs individuais foi avaliado de acordo com a ferramenta Risk of Bias 2.0 (RoB 2.0) da Colaboração Cochrane.^14^ A certeza geral da evidência e a força da recomendação foram analisadas conforme a abordagem desenvolvida pelo Grading of Recommendations Assessment, Development, and Evaluation (GRADE) Working Group.^15^

## Resultados

Os resultados da metanálise mostram que não houve diferença estatisticamente significativa nos desfechos de mortalidade geral e cardiovascular, com moderada certeza da evidência. Para qualidade de vida, a certeza da evidência foi baixa, também sem diferença significativa. Por outro lado, houve um aumento importante na incidência de hipercalemia, com alta certeza da evidência. Ainda assim, ao considerar o conjunto das evidências, o painel atribuiu maior peso aos benefícios observados nos desfechos clínicos considerados mais relevantes para os pacientes — como a redução de hospitalizações por insuficiência cardíaca e da piora clínica da condição —, avaliando que esses benefícios superam o risco identificado de hipercalemia. Destaca-se, contudo, que os valores e preferências dos pacientes devem ser incorporados ao processo de tomada de decisão.

### Sumário das evidências

**Figura 1:**
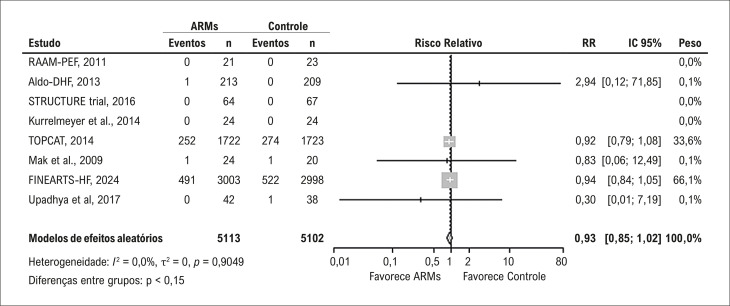
– Efeito do uso de antagonistas dos receptores de mineralocorticoides versus controle para o desfecho mortalidade geral. ARMs: antagonistas dos receptores de mineralocorticoides; IC: intervalo de confiança; RR: risco relativo. Fonte: elaboração própria.

Inicialmente, foi realizada uma busca por revisões sistemáticas, que resultou na identificação de 168 referências nas bases de dados. Após a remoção de 5 registros duplicados, 163 referências foram analisadas por título e resumo, das quais 13 foram selecionadas para leitura completa. Ao final dessa etapa, 2 referências foram incluídas^16,17^ (Figura 1S).

A avaliação do risco de viés dessas duas revisões sistemáticas, utilizando a ferramenta AMSTAR-2, revelou limitações metodológicas significativas em ambos os estudos (Tabela 1S). Diante disso, optou-se por conduzir uma nova busca por ECRs que avaliassem a eficácia e segurança dos ARMs em pacientes com ICFEp.

**Figura 2:**
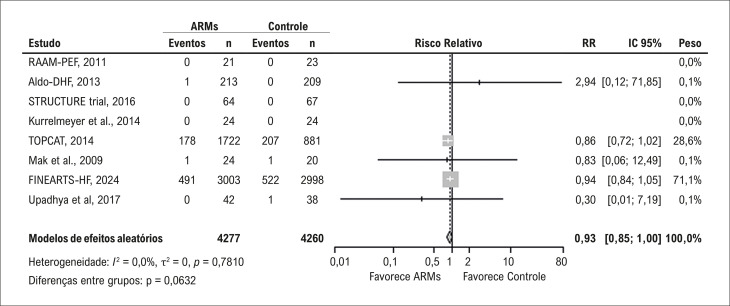
– Subanálise do efeito do uso de antagonistas dos receptores de mineralocorticoides versus controle para o desfecho mortalidade geral. ARMs: antagonistas dos receptores de mineralocorticoides; IC: intervalo de confiança; RR: risco relativo. Fonte: elaboração própria.

Na busca por ECRs, foram identificados 522 registros. Após a exclusão de 22 registros duplicados e 475 estudos com base em título e resumo, 27 referências foram selecionadas para leitura completa. Por fim, 8 estudos reportados em 11 publicações foram incluídos na revisão sistemática e metanálise (Figura 2S). As listas dos estudos excluídos e suas respectivas justificativas encontram-se detalhadas no material suplementar (Quadro 3S).

Dos 8 ECRs incluídos, 5 compararam espironolactona versus placebo, enquanto 2 avaliaram eplerenona versus placebo e 1 finerenona versus placebo.^4,5,8,18-26^ O tempo de seguimento dos estudos variou de 6 a 60 meses.

As características dos estudos incluídos e cujos resultados foram localizados estão apresentadas na Tabela 1.

**Tabela 1:** – Características dos ensaios clínicos randomizados incluídos (n = 8 ensaios, 11 referências)

Estudo (autor, ano)	Mak, 2009^22^	RAAM-PEF (Deswal, 2011)^24^	Aldo-DHF (Edelmann, 2013)^18^	Kurrelmeyer, 2014^20^	TOPCAT (Pitt, 2014, Pfeffer, 2015 e Lewis, 2016)^4,5,21^	STRUCTURE trial (Kosmala, 2016)^19^	Upadhya, 2017^23^	FINEARTS-HF (Solomon, 2024 e Cunningham, 2025)^8,25^
**Delineamento do estudo e registro do protocolo**	ECR paralelo, aberto NCT00505336	ECR paralelo, duplo-cego, fase IV NCT00108251	ECR paralelo, duplo-cego, fase IIb ISRCTN94726526	ECR paralelo, duplo-cego, fase IV NCT00206232	ECR paralelo, duplo-cego, fase III NCT00094302	ECR paralelo, duplo-cego, fase IV ACTRN12614000088640	ECR paralelo, duplo-cego, fase III NCT00123955	ECR paralelo, duplo-cego, fase III NCT04435626
**Local e período**	Irlanda Início em abril de 2006	Estados Unidos Início em agosto de 2006	Alemanha e Áustria (10 locais) Início em março 2007	Estados Unidos Início em julho de 2004	Estados Unidos, Canadá, Brasil, Argentina, Rússia e Geórgia Início em agosto de 2006	Polônia Início em novembro de 2011	Estados Unidos Início em abril de 2005	Estados Unidos, Argentina, Austrália, Áustria, Brasil, Bulgária, Canadá, China, Colômbia, República Tcheca, Dinamarca, Finlândia, Alemanha, Grécia, Hong Kong, Hungria, Índia, Israel, Itália, Japão, Coréia, Letônia, Lituânia, Malásia, México, Holanda, Nova Zelândia, Polônia, Portugal, Romênia, Rússia, Eslováquia, Espanha, Taiwan, Turquia, Ucrânia, Reino Unido Início em setembro de 2020
**Participantes**	Indivíduos de ambos os sexos com insuficiência cardíaca; fração de ejeção ≥ 45% (n = 44) Idade (média): 80 ± 7,8 anos Comorbidades: hipertensão (91%); diabetes mellitus (28%); hiperlipidemia (29%)	Indivíduos de ambos os sexos com insuficiência cardíaca; fração de ejeção ≥ 50% (n = 46) Idade (média): 70 ± 9 anos Comorbidades: hipertensão (100%); diabetes mellitus (61%); DAC (57%); fibrilação atrial (13,7%)	Indivíduos de ambos os sexos com insuficiência cardíaca; fração de ejeção ≥ 50% (n = 422) Idade (média): 67 ± 8 anos Comorbidades: hipertensão (92%); hiperlipidemia (65%); DAC (40%); diabetes mellitus (17%); fibrilação atrial (5%); DPOC (3%)	Indivíduos de ambos os sexos com insuficiência cardíaca; fração de ejeção ≥ 50% (n = 48) Idade (média): 71 ± 5 anos Comorbidades: hipertensão (83%); diabetes mellitus (38%); DAC (35%); fibrilação atrial (25%)	Indivíduos de ambos os sexos com insuficiência cardíaca; fração de ejeção ≥ 45% (n = 3.445) Idade (mediana): 68,7 anos Comorbidades: hipertensão (91,5%); dislipidemia (60,2%); DAC (58,8%); doença renal crônica (38,7%); diabetes mellitus (32,5%); fibrilação atrial (35,3%); infarto do miocárdio (26%); DPOC (11,7%); acidente vascular cerebral (7,7%)	Indivíduos de ambos os sexos com insuficiência cardíaca; fração de ejeção ≥ 50% (n = 150) Idade (média): 67 ± 8 anos Comorbidades: hipertensão (91,5%); diabetes mellitus (39,5%)	Indivíduos de ambos os sexos com insuficiência cardíaca; fração de ejeção ≥ 50% (n = 80) Idade (média): 71 ± 1 anos Comorbidades: hipertensão (87,5%); diabetes mellitus (23%); edema pulmonar (19%)	Indivíduos de ambos os sexos com insuficiência cardíaca; fração de ejeção ≥ 40% (n = 7.463) Idade (média): 72 ± 9,7 anos Comorbidades: hipertensão (88,8%); diabetes mellitus (40,7%); fibrilação atrial (38,2%)
**Intervenção**	Eplerenona 25 mg por dia por 6 meses, aumentada para 50 mg por dia de 6 a 12 meses (n = 24) 12 meses	Eplerenona 25 mg por dia por 2 semanas, aumentada para 50 mg por 22 semanas (n = 23) 6 meses	Espironolactona 25 mg por dia (n = 213) 12 meses	Espironolactona 25 mg por dia (n = 24) 6 meses	Espironolactona 15 mg 1 vez ao dia, que foi aumentado para um máximo de 45 mg ao dia durante os primeiros 4 meses (n = 1.722) 39,6 meses e 60 meses	Espironolactona 25 mg por dia (n = 75) 6 meses	Espironolactona 25 mg por dia (n = 42) 9 meses	Finerenona na dose máxima de 20 mg ou 40 mg 1 vez ao dia (n = 3.003) 32 meses
**Comparador**	Controle sem tratamento adicional (n = 20) 12 meses	Placebo (n = 23) 6 meses	Placebo (n = 209) 12 meses	Placebo (n = 24) 6 meses	Placebo (n = 1.723) 39,6 meses e 60 meses	Placebo (n = 75) 6 meses	Placebo (n = 38) 9 meses	Placebo (n = 2.998) 32 meses
**Desfechos e time points de avaliação**	Qualidade de vida, biomarcadores, função diastólica 12 meses	Capacidade funcional, qualidade de vida, biomarcadores, função diastólica, classe funcional 6,4 meses	Capacidade funcional, função diastólica, mortalidade geral, hospitalização, qualidade de vida, ansiedade, depressão, eventos adversos 12 meses	Capacidade funcional, função diastólica, biomarcadores, mortalidade geral, hospitalização por insuficiência cardíaca, hipercalemia e qualidade de vida 6 meses	Mortalidade geral, mortalidade cardiovascular, hospitalização, eventos adversos, hipercalemia e piora da função renal 39,6 meses Qualidade de vida 60 meses	Capacidade funcional, função diastólica, mortalidade geral, hospitalização, biomarcadores, eventos adversos 6 meses	Capacidade funcional, função diastólica, mortalidade, hospitalização, eventos adversos, aderência, qualidade de vida 9 meses	Mortalidade, hospitalização, piora da insuficiência cardíaca, qualidade de vida, eventos adversos, classe funcional 32 meses
**Fontes de financiamento**	St Vincent’s University Hospital, Ireland	Pfizer Pharmaceuticals	German-Austrian Heart Failure Study Group e German Competence Network of Heart Failure	Women’s Fund; Houston, Texas	National Heart, Lung, and Blood Institute, National Institutes of Health (HHSN268200425207C)	Wroclaw Medical University (ST-678) e Royal Hobart Hospital Foundation (13-024)	National Institutes of Health (NIH, R01AG18915); Claude D. Pepper Older Americans Independence Center of Wake Forest University (P30AG21332); the Clinical and Translational Science Institute of Wake Forest School of Medicine (NIH UL1TR001420); e o Kermit G. Phillips Chair in Cardiovascular Medicine of Wake Forest School of Medicine	Bayer

DAC: doença arterial coronária; DPOC: doença pulmonar obstrutiva crônica; ECR: ensaio clínico randomizado.

### Mortalidade geral

O uso de ARMs não resultou em diferença significativa no desfecho de mortalidade geral quando comparado ao grupo controle (RR: 0,93; intervalo de confiança de 95%: 0,85 a 1,02; n = 10.215; 8 ECRs; I^2^ = 0%; certeza de evidência moderada; Figura 1 e Tabela 2S). A certeza da evidência foi considerada moderada devido à imprecisão dos dados, com intervalo de confiança incluindo tanto uma redução de até 15% quanto um aumento de 2% no risco de mortalidade geral.

Foi realizada uma subanálise substituindo o estudo TOPCAT (2014) pelos dados da subpopulação das Américas, conforme descrito por Pfeffer e colaboradores (TOPCAT 2015),^5^ devido à significativa heterogeneidade entre as regiões participantes do estudo original. A decisão baseou-se na baixa taxa de eventos observada na coorte da Rússia/Geórgia, que compromete a validade externa dos resultados globais. A subpopulação das Américas apresentou características clínicas e resposta à intervenção mais compatíveis com os demais estudos incluídos na metanálise. Com essa substituição, o uso de ARMs continuou sem diferença significativa no desfecho de mortalidade geral em comparação ao grupo controle (RR: 0,91; intervalo de confiança de 95%: 0,83 a 1,00; n = 8.537; 8 ECRs; I^2^ = 0%; Figura 2), mantendo a certeza da evidência como moderada.

### Mortalidade cardiovascular

**Figura 3:**
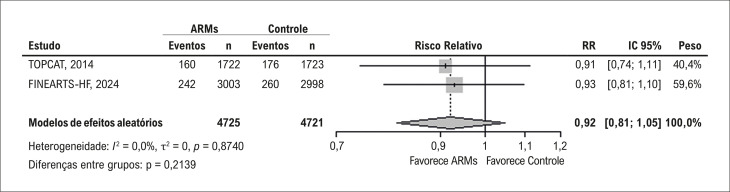
– Efeito do uso de antagonistas dos receptores de mineralocorticoides versus controle para o desfecho mortalidade cardiovascular. ARMs: antagonistas dos receptores de mineralocorticoides; IC: intervalo de confiança; RR: risco relativo. Fonte: elaboração própria.

O uso de ARMs não resultou em diferença significativa no desfecho mortalidade cardiovascular quando comparado ao grupo controle (RR: 0,92; intervalo de confiança de 95%: 0,81 a 1,05; n = 9.446; 2 ECRs; I^2^ = 0%; certeza de evidência moderada; Figura 3 e Tabela 2S). A certeza na evidência foi considerada moderada, com intervalo de confiança amplo, englobando desde uma redução de 19% até um aumento de 5% no risco, refletindo imprecisão nos achados.

**Figura 4:**
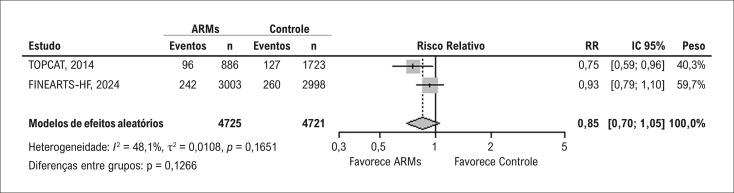
– Subanálise do efeito do uso de antagonistas dos receptores de mineralocorticoides versus controle para o desfecho mortalidade cardiovascular. ARMs: antagonistas dos receptores de mineralocorticoides; IC: intervalo de confiança; RR: risco relativo. Fonte: elaboração própria.

Na subanálise substituindo o estudo TOPCAT (2014) pelos dados da subpopulação das Américas, conforme descrito por Pfeffer e colaboradores (TOPCAT 2015),^5^ o uso de ARMs continuou sem diferença significativa no desfecho de mortalidade cardiovascular em comparação ao grupo controle (RR: 0,85; intervalo de confiança de 95%: 0,70 a 1,05; n = 7.768; 2 ECRs; I^2^ = 48,1%; Figura 4), mantendo a certeza da evidência como moderada.

### Hospitalização

**Figura 5:**
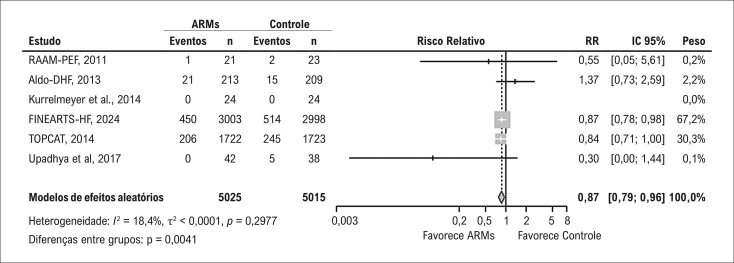
– Efeito do uso de antagonistas dos receptores de mineralocorticoides versus controle para o desfecho hospitalização por insuficiência cardíaca. ARMs: antagonistas dos receptores de mineralocorticoides; IC: intervalo de confiança; RR: risco relativo. Fonte: elaboração própria. Os dados do FINEARTS-HF são do efeito da finerenona comparado ao placebo em eventos iniciais de piora da insuficiência cardíaca.21

O uso de ARMs resultou em diferença no desfecho hospitalização cardiovascular quando comparado ao grupo controle (RR: 0,87; intervalo de confiança de 95%: 0,79 a 0,96; n = 10.040; 6 ECRs; I^2^ = 18,4%; certeza de evidência moderada; Figura 5 e Tabela 2S). A certeza na evidência foi considerada moderada, com o intervalo de confiança de 95% amplo, englobando uma redução de 4% a 21% no risco.

O estudo STRUCTURE avaliou 64 pacientes no grupo intervenção e 67 pacientes no grupo controle. Após 6 meses de tratamento, foram registradas 3 hospitalizações por causas cardíacas no grupo tratado com ARMs (4,7%) e 4 hospitalizações no grupo controle (6,0%). No entanto, os autores não detalharam os critérios diagnósticos ou clínicos utilizados para a definição de hospitalização por causas cardíacas e por isso o estudo não foi incluído nesta metanálise.

**Figura 6:**
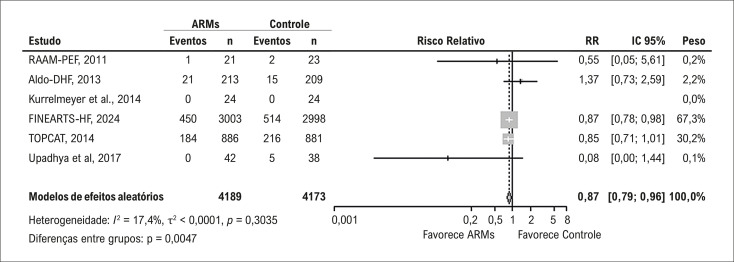
– Subanálise do efeito do uso de antagonistas dos receptores de mineralocorticoides versus controle para o desfecho hospitalização cardiovascular. ARMs: antagonistas dos receptores de mineralocorticoides; IC: intervalo de confiança; RR: risco relativo. Fonte: elaboração própria. Os dados do FINEARTS-HF são do efeito da finerenona comparado ao placebo em eventos iniciais de piora da insuficiência cardíaca.21

Na subanálise substituindo o estudo TOPCAT (2014) pelos dados da subpopulação das Américas, conforme descrito por Pfeffer et al. (TOPCAT 2015),^5^ o uso de ARMs continuou com a mesma diferença significativa no desfecho de hospitalização cardiovascular em comparação ao grupo controle (RR: 0,87; intervalo de confiança de 95%: 0,79 a 0,96; n = 8.362; 6 ECRs; I^2^ = 17,4%; Figura 6), mantendo a certeza da evidência como moderada.

### Piora da insuficiência cardíaca

**Figura 7:**
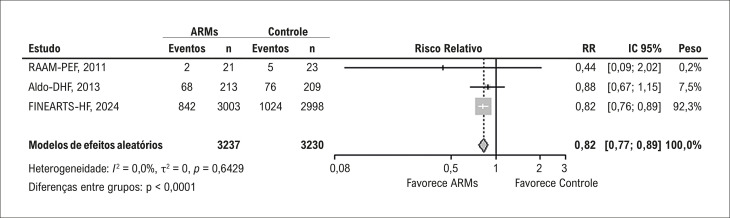
– Efeito do uso de antagonistas dos receptores de mineralocorticoides versus controle para o desfecho piora da insuficiência cardíaca. ARMs: antagonistas dos receptores de mineralocorticoides; IC: intervalo de confiança; RR: risco relativo. Fonte: elaboração própria.

O uso de ARMs resultou na redução em 18% do risco de piora da insuficiência cardíaca quando comparado ao grupo controle (RR: 0,82; intervalo de confiança de 95%: 0,77 a 0,89; n = 6.467; 3 ECRs; I^2^ = 0%; certeza de evidência alta; Figura 7 e Tabela 2S). Foi realizada uma análise de sensibilidade com a exclusão do estudo Aldo-DHF, devido à população com insuficiência cardíaca relativamente estável (NT-proBNP não elevado). Nessa análise, o uso de ARMs manteve a redução significativa do risco (RR: 0,82; intervalo de confiança de 95%: 0,76 a 0,88; 2 ECRs; n = 6.045), reforçando a robustez dos achados.

Os estudos incluídos adotaram definições variáveis para caracterizar a piora da insuficiência cardíaca. O estudo RAAM-PEF (2011) definiu a piora como hospitalização por insuficiência cardíaca ou necessidade de intensificação do tratamento, incluindo administração de diuréticos intravenosos ou aumento da dose de diuréticos orais. O estudo Aldo-DHF (2013) considerou como critérios de piora a intensificação da dispneia, bem como o agravamento ou surgimento de edema. Já o estudo FINEARTS-HF (2024) definiu a piora da insuficiência cardíaca como a ocorrência de hospitalização não planejada, seja inicial ou recorrente, ou visita urgente devido à condição.

### Qualidade de vida

Esse desfecho foi avaliado através das ferramentas Minnesota Living With Heart Failure Questionnaire (MLHFQ), Kansas City Cardiomyopathy Questionnaire (KCCQ), EQ5D Visual analog scale (VAS) e 36-Item Short Form Health Survey (SF-36).

**Figura 8:**
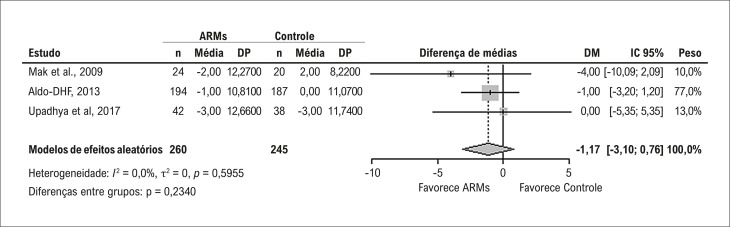
– Diferença de média entre o uso de antagonistas dos receptores de mineralocorticoides versus controle para o desfecho qualidade de vida avaliado pela ferramenta MLHFQ. ARMs: antagonistas dos receptores de mineralocorticoides; DM: diferença de médias; DP: desvio padrão; IC: intervalo de confiança. Fonte: elaboração própria.

Na análise dos estudos que utilizaram a ferramenta MLHFQ, o uso de ARMs não resultou em diferença significativa no desfecho de qualidade de vida em comparação ao grupo controle (DM: −1,17; intervalo de confiança de 95%: −3,10 a 0,76; n = 505; 3 ECRs; I^2^ = 0%; certeza da evidência moderada; Figura 8 e Tabela 2S). A certeza da evidência foi considerada moderada devido à imprecisão dos dados, com intervalo de confiança que varia desde uma possível melhora discreta (3,1 pontos) até uma piora mínima (0,76 pontos). Adicionalmente, o estudo de Upadhya de 2017^23^ avaliou os domínios emocional e físico do MLHFQ, e também não foram observadas diferenças significativas entre o grupo que utilizou espironolactona e o grupo placebo após 6 meses de tratamento.

**Figura 9:**
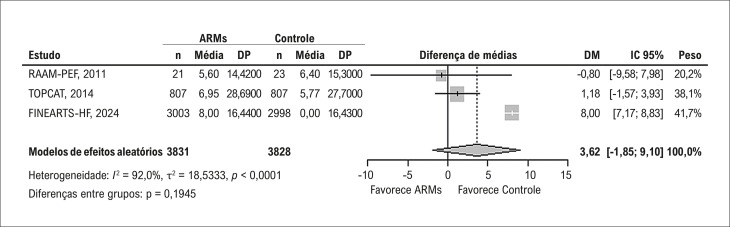
– Diferença de média entre o uso de antagonistas dos receptores de mineralocorticoides versus controle para o desfecho qualidade de vida avaliado pela ferramenta KCCQ Overall Summary Score. ARMs: antagonistas dos receptores de mineralocorticoides; DM: diferença de médias; DP: desvio padrão; IC: intervalo de confiança. Fonte: elaboração própria.

**Figura 10:**
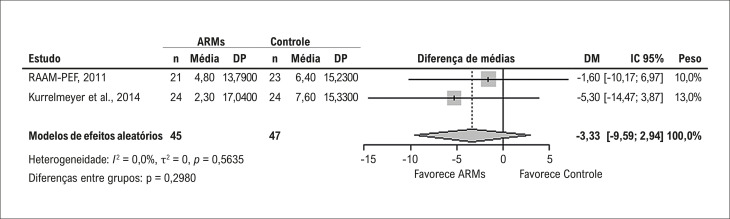
– Diferença de média entre o uso de antagonistas dos receptores de mineralocorticoides versus controle para o desfecho qualidade de vida avaliado pela ferramenta KCCQ Clinical Summary Score. ARMs: antagonistas dos receptores de mineralocorticoides; DM: diferença de médias; DP: desvio padrão; IC: intervalo de confiança. Fonte: elaboração própria.

Entre os estudos que utilizaram a ferramenta KCCQ Overall Summary Score, também não foi observada diferença significativa entre os grupos (DM: 3,62; intervalo de confiança de 95%: −1,85 a 9,10; n = 7.659; 3 ECRs; I^2^ = 92%; certeza da evidência moderada; Figura 9 e Tabela 2S). A certeza na evidência foi considerada moderada devido à imprecisão, com intervalo de confiança amplo, que inclui desde uma piora discreta (−1,85 pontos) até uma melhora modesta (9,10 pontos). Resultados semelhantes foram observados no Clinical Summary Score (DM: −3,33; intervalo de confiança de 95%: −9,59 a 2,94; 2 ECRs, n = 92; I^2^ = 0%; Figura 10 e Tabela 2S), porém com certeza baixa devido ao pequeno tamanho amostral e imprecisão dos resultados, onde o intervalo sugeriu desde possível piora (−9,59) até benefício (2,94) (Tabela 2S).

O estudo TOPCAT de 2016 também avaliou a qualidade de vida por meio da ferramenta EQ5D VAS. Não foi observada diferença estatisticamente significativa na variação média do escore entre os grupos tratados com espironolactona (variação média: 9,04; erro padrão: 0,96) e placebo (variação média: 8,67; erro padrão: 0,86) ao longo de 60 meses de acompanhamento.

No estudo Aldo-DHF de 2013, o uso de espironolactona não resultou em melhora significativa no desfecho de qualidade de vida avaliados pelas escalas SF-36 Physical Functioning (diferença ajustada de 1 ponto; intervalo de confiança de 95%: −2 a 4; p = 0,62) e SF-36 Global Self-Assessment (diferença ajustada de 0,0; intervalo de confiança de 95%: −0,1 a 0,1; p = 0,79), quando comparado ao grupo placebo após 12 meses de tratamento.

### Hipercalemia

**Figura 11:**
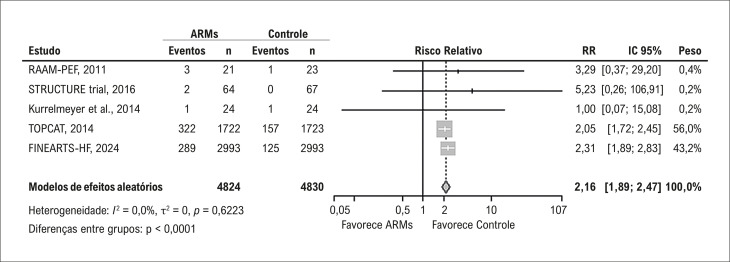
– Efeito do uso de antagonistas dos receptores de mineralocorticoides versus controle para o desfecho de hipercalemia. ARMs: antagonistas dos receptores de mineralocorticoides; IC: intervalo de confiança; RR: risco relativo. Fonte: elaboração própria.

Os resultados demonstraram que o uso de ARMs aumentou o risco de hipercalemia em comparação ao grupo controle (RR: 2,16; intervalo de confiança de 95%: 1,89 a 2,47; n = 9.654; 5 ECRs; I^2^ = 0%; certeza de evidência alta; Figura 11 e Tabela 2S). O intervalo de confiança indicou que o tratamento com ARM aumentou o risco de hipercalemia, com uma variação estimada entre 89% e 147% de aumento.

**Figura 12:**
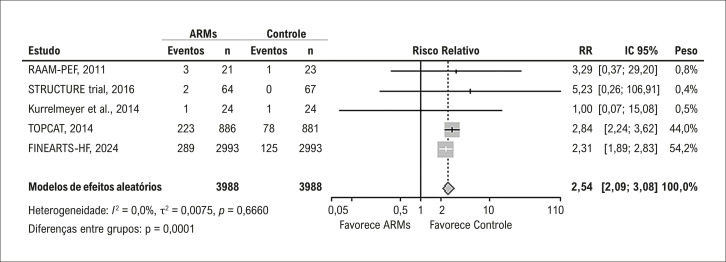
– Subanálise do efeito do uso de antagonistas dos receptores de mineralocorticoides versus controle para o desfecho de hipercalemia. ARMs: antagonistas dos receptores de mineralocorticoides; IC: intervalo de confiança; RR: risco relativo. Fonte: elaboração própria.

Na subanálise substituindo o estudo TOPCAT (2014) pelos dados da subpopulação das Américas, conforme descrito por Pfeffer e colaboradores (TOPCAT 2015),^5^ o uso de ARMs continuou com o aumento de risco de hipercalemia em comparação ao grupo controle (RR: 2,54; intervalo de confiança de 95%: 2,09 a 3,08; n = 7.976; 5 ECRs; I^2^ = 0%), mantendo a certeza da evidência como moderada (Figura 12).

### Deterioração da função renal

**Figura 13:**
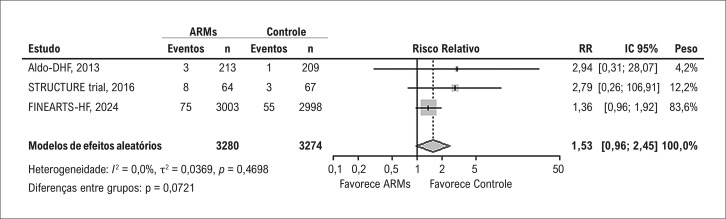
– Efeito do uso de antagonistas dos receptores de mineralocorticoides versus controle para o desfecho deterioração da função renal. ARMs: antagonistas dos receptores de mineralocorticoides; IC: intervalo de confiança; RR: risco relativo. Fonte: elaboração própria.

A análise revelou que o uso de ARMs apresentou tendência à piora da função renal em comparação ao controle (RR: 1,53; intervalo de confiança de 95%: 0,96 a 2,45; n = 6.554; 3 ECRs; I^2^ = 0%; certeza de evidência moderada; Figura 13 e Tabela 2S). A certeza na evidência foi considerada moderada devido à imprecisão dos dados visto que, embora o RR sugira um aumento potencial de 53%, o intervalo de confiança amplo engloba desde uma redução marginal de 4% até um aumento expressivo de 145%.

### Risco de viés e certeza da evidência

A avaliação do risco de viés dos ECRs foi realizada para todos os desfechos considerados. Os estudos Aldo-DHF, TOPCAT (2014) e FINEARTS-HF (2024) não apresentaram viés em nenhum dos domínios avaliados, correspondendo a 34% do total. Das 9 publicações incluídas, 4 (44%) foram classificadas como de alto risco de viés, enquanto 2 estudos (22%) apresentaram algumas preocupações quanto ao risco de viés em sua avaliação global. O material suplementar (Figura 3S) apresenta o quadro com os detalhes dos julgamentos realizados para cada domínio da ferramenta.

A certeza no conjunto final das evidências foi avaliada por meio da abordagem GRADE para todos os desfechos da comparação, sendo apenas considerado os *time points* mais longos de acompanhamento nos estudos e a amostra geral (Tabela 2 e Tabela 2S).

**Tabela 2:** – Avaliação da certeza no conjunto final das evidências

População: Pacientes com insuficiência cardíaca com fração de ejeção preservada Intervenção: ARMs Comparação: Controle						
Desfechos	Efeitos absolutos potenciais (IC 95%)		Efeito relativo (IC 95%)	№ de participantes (estudos)	Certeza da evidência (GRADE)	Declarações narrativas
	Risco com controle	Risco com ARMs				
**Mortalidade geral** Seguimento: variação 6 meses para 39,6 meses	156 por 1.000	**145 por 1.000** (132 a 159)	**RR 0,93** (0,85 a 1,02)	10.234 (8 ECRs)	⨁⨁⨁◯ Moderada^a,b^	ARMs provavelmente não resultam em diferença de risco na mortalidade geral.
**Mortalidade cardiovascular** Seguimento: variação 32 meses para 39,6 meses	85 por 1.000	**78 por 1.000** (69 a 89)	**RR 0,92** (0,81 a 1,05)	9.446 (2 ECRs)	⨁⨁⨁◯ Moderada^b^	ARMs provavelmente não resultam em diferença de risco na mortalidade cardiovascular.
**Hospitalização por insuficiência** cardíaca Seguimento: variação 6 meses para 39,6 meses	156 por 1.000	**135 por 1.000** (123 a 150)	**RR 0,87** (0,79 a 0,96)	10.040 (6 ECRs)	⨁⨁⨁⨁ Alta	ARMs resultam em redução no risco de hospitalização por insuficiência cardíaca.
**Piora da insuficiência cardíaca**	342 por 1.000	**281 por 1.000** (263 a 304)	**RR 0,82** (0,77 a 0,89)	6.467 (3 ECRs)	⨁⨁⨁⨁ Alta	ARMs reduzem o risco da piora da insuficiência cardíaca.
**Qualidade de vida** (avaliada com MLHFQ) Seguimento: variação 9 meses para 12 meses	A média qualidade de vida foi 0	**DM 1,17 menor** (3,10 menor a 0,76 mais alto)	-	505 (3 ECRs)	⨁⨁◯◯ Baixa^b,c^	ARMs podem não resultar em diferença na qualidade de vida quando avaliada pelo MLHFQ.
**Qualidade de vida** (avaliada com KCCQ Overall Summary Score) Seguimento: variação 6 meses para 39,6 meses	-	**DM 3,62 mais alto** (1,85 menor a 9,10 mais alto)	-	7.659 (3 ECRs)	⨁⨁◯◯ Baixa^b,d^	ARMs podem não resultar em diferença na qualidade de vida quando avaliada pelo KCCQ.
**Hipercalemia** Seguimento: variação 6 meses para 39,6 meses	59 por 1.000	**127 por 1.000** (111 a 128)	**RR 2,16** (1,89 a 2,47)	9.654 (5 ECRs)	⨁⨁⨁⨁ Alta	ARMs aumentam o risco da hipercalemia.
**Deterioração da função renal** Seguimento: variação 6 meses para 32 meses	18 por 1.000	**28 por 1.000** (17 a 44)	**RR 1,53** (0,96 a 2,45)	6.554 (3 ECRs)	⨁⨁⨁◯ Moderada^a,b^	ARMs provavelmente resultam na deterioração da função renal.

ARM: antagonista dos receptores de mineralocorticoides; ECR: ensaio clínico randomizado; IC: intervalo de confiança; RR: risco relativo. O MLHFQ (Minnesota Living with Heart Failure Questionnaire) possui pontuação variando de 0 a 105, onde quanto maior a pontuação, pior a qualidade de vida. O KCCQ (Kansas City Cardiomyopathy Questionnaire) possui pontuação variando de 0 a 100 em cada domínio, onde quanto maior a pontuação, melhor a qualidade de vida. Explicações: a. Limitações metodológicas: Embora alguns estudos tenham apresentado risco de viés, os que mais influenciaram a análise foram classificados como de baixo risco de viés. b. Imprecisão: Intervalo de confiança amplo, o que indica uma imprecisão nos resultados. c. Limitações metodológicas: De acordo com a ferramenta RoB 2, o estudo Mak (2009)^22^ foi classificado com algumas preocupações, com penalizações nos domínios processo de randomização e relato seletivo dos desfechos. Já o estudo Upadhya (2017)23 foi avaliado como tendo algumas preocupações com penalizações nos domínios processo de randomização e desvios nas intervenções pretendidas. d. Limitações metodológicas: De acordo com a ferramenta RoB 2, os estudos RAAM-PEF (2011)^24^ e TOPCAT (2016)^21^ foram classificados como de alto risco de viés, com penalizações em diversos domínios, comprometendo a confiabilidade da estimativa de efeito. N pequeno. Fonte: elaboração própria.

## Discussão

Esta revisão sistemática avaliou a eficácia e segurança dos ARMs em pacientes com ICFEp e ICFElr, demonstrando benefício clínico consistente na evolução da doença. Embora o impacto sobre a mortalidade tenha sido limitado, observou-se uma redução estatisticamente significativa nas hospitalizações por insuficiência cardíaca, indicando um efeito relevante sobre a morbidade dos pacientes. Esse benefício se apresentou tanto para a espironolactona quanto para a finerenona.

Vale ressaltar que na publicação original dos resultados do estudo TOPCAT, a análise do componente do desfecho primário de hospitalizações por insuficiência cardíaca também demonstrou benefício da espironolactona, com *hazard ratio*: 0,83 (intervalo de confiança de 95%: 0,69 a 0,99; p = 0,04). Da mesma forma, ainda que o estudo FINEARTS-HF tenha atingido redução estatisticamente significativa na ocorrência do desfecho primário composto por morte cardiovascular ou eventos de piora de insuficiência cardíaca (primeira ou recorrente hospitalização não planejada por insuficiência cardíaca ou visita à urgência por insuficiência cardíaca), apenas o componente do desfecho primário de piora da insuficiência cardíaca mostrou redução significativa com o uso de finerenona (*hazard ratio*: 0,82; intervalo de confiança de 95%: 0,71 a 0,94; p = 0,006), com impacto neutro na morte cardiovascular (*hazard ratio*: 0,93; intervalo de confiança de 95%: 0,78 a 1,11).

Esses resultados também são semelhantes aos de outros estudos testando tratamentos em pacientes com ICFEp ou ICFElr, mostrando benefícios na redução de hospitalizações ou eventos de insuficiência cardíaca, mas sem impacto na mortalidade geral ou mortalidade cardiovascular, como foi observado nos estudos com inibidores de SGLT2.^26,27^ Várias análises têm sugerido que a falta de impacto nos desfechos de mortalidade na ICFEp ou ICFElr se deva às características particulares dessa população, como idade avançada e múltiplas comorbidades, fazendo com que a principal causa de morte seja não cardiovascular.^28^

Em relação à qualidade de vida, não foram identificadas diferenças estatisticamente significativas entre os grupos nas diversas escalas avaliadas (MLHFQ, KCCQ, EQ5D-VAS e SF-36). Tais resultados podem sugerir que os ARMs têm pouco impacto no bem-estar percebido pelos pacientes, mesmo quando há melhora em outros parâmetros clínicos. Essa ausência de efeito pode ser atribuída às variações nos períodos de acompanhamento e às diferenças nas características clínicas dos pacientes entre os estudos incluídos.

Do ponto de vista da segurança, a terapia com ARMs esteve associada a importantes eventos adversos, particularmente um aumento significativo no risco de hipercalemia (com alta certeza de evidência) e uma tendência à deterioração da função renal (com moderada certeza de evidência). Esses achados ressaltam a necessidade de uma cuidadosa avaliação individualizada da relação risco-benefício, especialmente em populações com maior predisposição a distúrbios eletrolíticos e renais (por exemplo, idosos e portadores de doença renal crônica). Por outro lado, a rigorosa monitoração dos níveis de creatinina e potássio sérico deve fazer parte do manejo clínico desses pacientes.^29^

A presente revisão sistemática seguiu uma metodologia rigorosa e transparente, com estratégias de busca amplas e sensíveis em múltiplas bases de dados, visando à identificação dos estudos relevantes. A seleção de textos completos e a extração dos dados foram realizadas de forma independente por revisores, reduzindo o risco de viés na condução do estudo. A avaliação da qualidade

metodológica dos ECRs foi conduzida com a ferramenta RoB 2.0, que permitiu uma análise detalhada dos possíveis riscos de viés em diferentes domínios, com avaliação por desfecho. Além disso, a certeza da evidência foi classificada de acordo com a abordagem GRADE, o que reforça a confiança nos achados e contribui para uma tomada de decisão clínica mais informada e baseada em evidências de qualidade.

Entre as limitações deste estudo, destaca-se a falta de padronização na avaliação de alguns desfechos, como qualidade de vida e eventos de piora da insuficiência cardíaca, o que dificulta a comparação direta entre os estudos. Ainda, por se tratar de uma revisão rápida, adotou-se triagem realizada por um revisor com checagem por um segundo, em vez da dupla revisão independente, o que pode aumentar o risco de vieses na seleção e extração dos dados.

## Conclusão

Os resultados desta metanálise dão apoio à recomendação de emprego dos ARMs, espironolactona ou finerenona, em pacientes com ICFEp ou ICFElr, para redução das hospitalizações ou eventos de insuficiência cardíaca (Figura Central).

## Material suplementar

Material Suplementar
