# Supplementary material for: Revisão Sistemática: Antagonistas dos Receptores de Mineralocorticoides na Insuficiência Cardíaca com Fração de Ejeção Preservada e Levemente Reduzida
Source: Arq Bras Cardiol. 2026 May 26;123(4):e20250622. [Article in Portuguese] doi: 10.36660/abc.20250622 (PMC13398873; doi:10.36660/abc.20250622)
Supplement: Material Suplementar [file 0066-782x-abc-123-4-e20250622-suppl01.pdf]

Material Suplementar

Sumário

Quadro 1S. Pergunta estruturada, acrônimo PICOS.

Quadro 2S. Estratégia de busca bibliográfica para revisões sistemáticas realizada em 26 de fevereiro de 2025.

Figura 1S. Fluxograma PRISMA da busca por revisões sistemáticas

Tabela 1S. Características das revisões sistemáticas selecionadas.

Quadro 3S. Estratégia de busca bibliográfica para ensaios clínicos randomizados

Figura 2S. Fluxograma PRISMA da busca por ensaios clínicos randomizados

Quadro 3S. Estudos excluídos e motivos de exclusão

Figura 3S. Avaliação da qualidade metodológica utilizando a ferramenta Risk of Bias 2.0.

Tabela 2S. Tabela resumo de evidências para avaliação de eficácia e segurança dos antagonistas dos receptores de mineralocorticóides comparado ao controle para tratamento de pacientes com insuficiência cardíaca com fração de ejeção preservada

Quadro 1S. Pergunta estruturada, acrônimo PICOS.

**Acrônimo**	**Definições**
P (população)	Pacientes com insuficiência cardíaca com fração de ejeção preservada (ICFEP), com fração de ejeção ≥ 40%, sintomáticos nas classes II, III ou IV da NYHA. Serão excluídos pacientes com taxa de filtração glomerular < 25 ml/min/1.73 m² ou com níveis de potássio sérico > 5,0 mmol/l.
I (intervenção)	Uso de antagonista mineralocorticoide (mra), como finerenona (20 mg ou 40 mg), espironolactona (25 mg ou 50 mg) ou eplerenona (25 mg ou 50 mg). A eplerenona será considerada, mesmo que não existam ECRs específicos para ICFEP com esse medicamento.
C (comparador(es))	Placebo ou tratamento padrão, onde pacientes no braço placebo estariam recebendo o tratamento usual (standard of care) para ICFEP.
O (desfechos – *outcomes*)	Mortalidade cardiovascular, mortalidade geral, hospitalização por insuficiência cardíaca, qualidade de vida, hipercalemia, deterioração da função renal.
S (tipo de estudo – *study design*)	Revisão sistemática de ensaios clínicos randomizados (ECRs) ou análise de ECRs individuais.

Fonte: elaboração própria

Quadro 2S. Estratégia de busca bibliográfica para revisões sistemáticas realizada em 26 de fevereiro de 2025.

Base	Estratégia de busca	Registros recuperados
Medline	#1 "Mineralocorticoid Receptor Antagonists"[Mesh] OR "Mineralocorticoid Receptor Antagonists" OR (Antagonists, Mineralocorticoid Receptor) OR (Receptor Antagonists, Mineralocorticoid) OR (Mineralocorticoid Receptor Antagonist) OR (Antagonist, Mineralocorticoid Receptor) OR (Receptor Antagonist, Mineralocorticoid) OR (Aldosterone Receptor Antagonists) OR (Antagonists, Aldosterone Receptor) OR (Receptor Antagonists,	90

	Aldosterone) OR (Aldosterone Receptor Antagonist) OR (Antagonist, Aldosterone Receptor) OR (Receptor Antagonist, Aldosterone) OR (Mineralocorticoid Antagonist) OR (Antagonist, Mineralocorticoid) OR (Mineralocorticoid Antagonists) OR (Antagonists, Mineralocorticoid) OR (Aldosterone Antagonists) OR (Antagonists, Aldosterone) OR (Aldosterone Antagonist) OR (Antagonist, Aldosterone)<br>#2 "Mineralocorticoid Receptor Antagonists" [Pharmacological Action] OR (` drospirenone (Supplementary Concept)) OR (RU 28318 (Supplementary Concept)) OR (N-(3-(1-cyclopropyl-1-(2,4-difluorophenyl)ethyl)-1H-indol-7-yl)methanesulfonamide (Supplementary Concept)) OR (de-O-methyllasiodiplodin (Supplementary Concept)) OR (N-4,4-dimethyl-2-thioxo-1,4-dihydro-2H-3,1-benzoxazin-6-ylthiophene-2-sulfonamide (Supplementary Concept)) OR (6-(1-(4-fluoro-2-methylphenyl)-3-(trifluoromethyl)-1H-pyrazol-5-yl)-2H-1,4-benzoxazin-3(4H)-one (Supplementary Concept)) OR ((("Canrenoic Acid"[Mesh]) OR "Canrenone"[Mesh]) OR "Spironolactone"[Mesh]) OR "Eplerenone"[Mesh]<br>#3 (Soludactone) OR (Soldactone) OR (Spiroctan) OR (Aldadiene) OR (Phanurane) OR (Verospirone) OR (Spirolactone) OR (Veroshpiron) OR (Aldactone) OR (Aldactone A) OR (Spirolang) OR (Aquareduct) OR (Duraspiron) OR (Espironolactona Alter) OR (Espironolactona Mundogen) OR (Flumach) OR (Frumikal) OR (Jenaspiron) OR (Novo-Spiroton) OR (NovoSpiroton) OR (Novo Spiroton) OR (Practon) OR (Spiractin) OR (Spiro L.U.T.) OR (Spiro Von Ct) OR (Ct, Spiro Von) OR (Von Ct, Spiro) OR (Spirobeta) OR (Spirogamma) OR (Spironone) OR (Spirospare) OR (Verospiron) OR (Spirono-Isis) OR (Spirono Isis) OR (Eplerenon) OR (Inspra)<br><br>#4 #1 OR #2 OR #3<br><br>#5 "Heart Failure, Diastolic"[Mesh] OR (Diastolic Heart Failures) OR (Diastolic Heart Failure) OR (Heart Failure, Preserved Ejection	

	Fraction) OR (Heart Failure, Normal Ejection Fraction) OR (Preserved Ejection Fraction Heart Failure) OR (Normal Ejection Fraction Heart Failure)<br><br>#6((“Meta-Analysis as Topic”[MeSH] OR meta analy*[TIAB] OR metaanaly*[TIAB] OR “MetaAnalysis”[PT] OR “Systematic Review”[PT] OR “Systematic Reviews as Topic”[MeSH] OR systematic review*[TIAB] OR systematic overview*[TIAB] OR “Review Literature as Topic”[MeSH]) OR (cochrane[TIAB] OR embase[TIAB] OR psychlit[TIAB] OR psyclit[TIAB] OR psychinfo[TIAB] OR psycinfo[TIAB] OR cinahl[TIAB] OR cinhal[TIAB] OR “science citation index”[TIAB] OR bids[TIAB] OR cancerlit[TIAB]) OR (reference list*[TIAB] OR bibliograph*[TIAB] OR hand-search*[TIAB] OR “relevant journals”[TIAB] OR manual search*[TIAB]) OR ((“selection criteria”[TIAB] OR “data extraction”[TIAB]) AND “Review”[PT])) NOT (“Comment”[PT] OR “Letter”[PT] OR “Editorial”[PT] OR (“Animals”[MeSH] NOT (“Animals”[MeSH] AND “Humans”[MeSH])))<br><br>#4 AND #5 AND #6	
EMBASE	#1 'antimineralocorticoid'/exp OR 'antimineralocorticoid' OR 'mineralocorticoid receptor antagonists'/exp OR 'mineralocorticoid receptor antagonists' OR 'mineralocorticoid antagonist'/exp OR 'mineralocorticoid antagonist' OR 'aldosterone antagonism'/exp OR 'aldosterone antagonism' OR 'aldosterone antagonists'/exp OR 'aldosterone antagonists' OR 'anti aldosterone'/exp OR 'anti aldosterone' OR 'antialdosterone'/exp OR 'antialdosterone' OR 'antialdosterone agent'/exp OR 'antialdosterone agent' OR 'selective aldosterone receptor antagonist'/exp OR 'selective aldosterone receptor antagonist' OR 'aldosterone antagonist'/exp OR 'aldosterone antagonist'	74

	#2 'diastolic dysfunction'/exp OR 'diastolic dysfunction' OR 'hf with preserved ejection fraction'/exp OR 'hf with preserved ejection fraction' OR 'hf-pef (heart failure with preserved ejection fraction)'/exp OR 'hf-pef (heart failure with preserved ejection fraction)' OR 'hfpef (heart failure with preserved ejection fraction)'/exp OR 'hfpef (heart failure with preserved ejection fraction)' OR 'heart failure with preserved ejection fraction'/exp OR 'heart failure with preserved ejection fraction'<br><br>#3 ('systematic review' OR 'meta-analysis') AND [review]/lim OR 'meta analysis'/exp OR 'meta analysis' OR 'systematic review'/exp OR 'systematic review' OR 'systematic review (topic)'/exp OR 'systematic review (topic)' OR 'meta analysis (topic)'/exp OR 'meta analysis (topic)' OR 'biomedical technology assessment'/exp OR 'biomedical technology assessment' OR 'network meta-analysis'/exp OR 'network meta-analysis' OR ((systematic* NEAR/3 (review* OR overview*)):ti,ab,kw) OR ((methodologic* NEAR/3 (review* OR overview*)):ti,ab,kw) OR ((quantitative NEAR/3 (review* OR overview* OR synthes*)):ti,ab,kw) OR ((research NEAR/3 (integrati* OR overview*)):ti,ab,kw) OR ((integrative NEAR/3 (review* OR overview*)):ti,ab,kw) OR ((collaborative NEAR/3 (review* OR overview*)):ti,ab,kw) OR ((pool* NEAR/3 analy*):ti,ab,kw) OR 'data synthes*':ti,ab,kw OR 'data extraction*':ti,ab,kw OR 'data abstraction*':ti,ab,kw OR 'handsearch*':ti,ab,kw OR 'hand search*':ti,ab,kw OR 'mantel haenszel':ti,ab,kw OR 'peto':ti,ab,kw OR 'der simonian':ti,ab,kw OR 'dersimonian':ti,ab,kw OR 'fixed effect*':ti,ab,kw OR 'latin square*':ti,ab,kw OR 'met analy*':ti,ab,kw OR 'metanaly*':ti,ab,kw OR 'technology assessment*':ti,ab,kw OR 'hta':ti,ab,kw OR 'htas':ti,ab,kw OR 'technology overview*':ti,ab,kw OR 'technology appraisal*':ti,ab,kw OR 'meta regression*':ti,ab,kw OR 'metaregression*':ti,ab,kw OR 'meta-analy*':ti,ab,kw,ok OR 'metaanaly*':ti,ab,kw,ok OR 'systematic review*':ti,ab,kw,ok OR 'biomedical technology assessment*':ti,ab,kw,ok OR 'bio-medical	

	technology assessment*':ti,ab,kw,ok OR medline:ti,ab,ok OR cochrane:ti,ab,ok OR pubmed:ti,ab,ok OR medlars:ti,ab,ok OR embase:ti,ab,ok OR cinahl:ti,ab,ok OR cochrane OR 'health near/2 technology assessment' OR 'evidence report' OR ((comparative NEAR/3 (efficacy OR effectiveness)):ti,ab,kw,ok) OR 'outcomes research':ti,ab,kw,ok OR 'relative effectiveness':ti,ab,kw,ok OR ((('indirect' OR 'indirect treatment' OR 'mixed-treatment' OR 'bayesian') NEAR/3 comparison*):ti,ab,kw,ok) OR 'meta-analysis'/dm OR 'systematic review'/dm OR ((multi* NEAR/3 treatment NEAR/3 comparison*):ti,ab,kw,ok) OR ((mixed NEAR/3 treatment NEAR/3 ('meta analy*' OR metaanaly*)):ti,ab,kw,ok) OR (umbrella:ti,ab,kw,ok AND review*:ti,ab,kw,ok) OR ((multi* NEAR/2 paramet* NEAR/2 evidence NEAR/2 synthesis):ti,ab,kw,ok) OR ((multiparamet* NEAR/2 evidence NEAR/2 synthesis):ti,ab,kw,ok) OR (('multi paramet*' NEAR/2 evidence NEAR/2 synthesis):ti,ab,kw,ok)<br><br>#4 [embase]/lim NOT ([embase]/lim AND [medline]/lim)<br><br>#1 AND #2 AND #3 AND #4	
COCHRANE (CENTRAL)	#1 MeSH descriptor: [Mineralocorticoid Receptor Antagonists] explode all trees<br>#2 “Aldosterone Antagonist” OR “Aldosterone Antagonists” OR “Antagonists, Aldosterone” OR “Antagonist, Aldosterone” OR “Receptor Antagonists, Mineralocorticoid” OR “ ntagonist, Aldosterone Receptor” OR “Receptor Antagonists, Aldosterone” OR “Aldosterone Receptor Antagonists” OR “Receptor Antagonist, Mineralocorticoid” OR “Antagonist, Mineralocorticoid Receptor” OR “Mineralocorticoid Receptor Antagonist” OR “Receptor Antagonist, Aldosterone” OR “Aldosterone Receptor Antagonist” OR “Mineralocorticoid Antagonist” OR “Antagonists, Mineralocorticoid” OR “Antagonist, Mineralocorticoid” OR “Mineralocorticoid Antagonists” OR “Antagonists, Aldosterone	4

	Receptor” OR “Antagonists, Mineralocorticoid Receptor” 1121<br>#3 MeSH descriptor: [Heart Failure, Diastolic] explode all trees<br>#4 “Heart Failure, Normal Ejection Fraction” OR “Diastolic Heart Failures” OR “Diastolic Heart Failure” OR “Heart Failure, Preserved Ejection Fraction” OR “Preserved Ejection Fraction Heart Failure”<br>#5 #1 OR #2<br>#6 #3 OR #4<br>#7 #5 AND #6	
Total		168

Fonte: elaboração própria

Figura 1S. Fluxograma PRISMA da busca por revisões sistemáticas

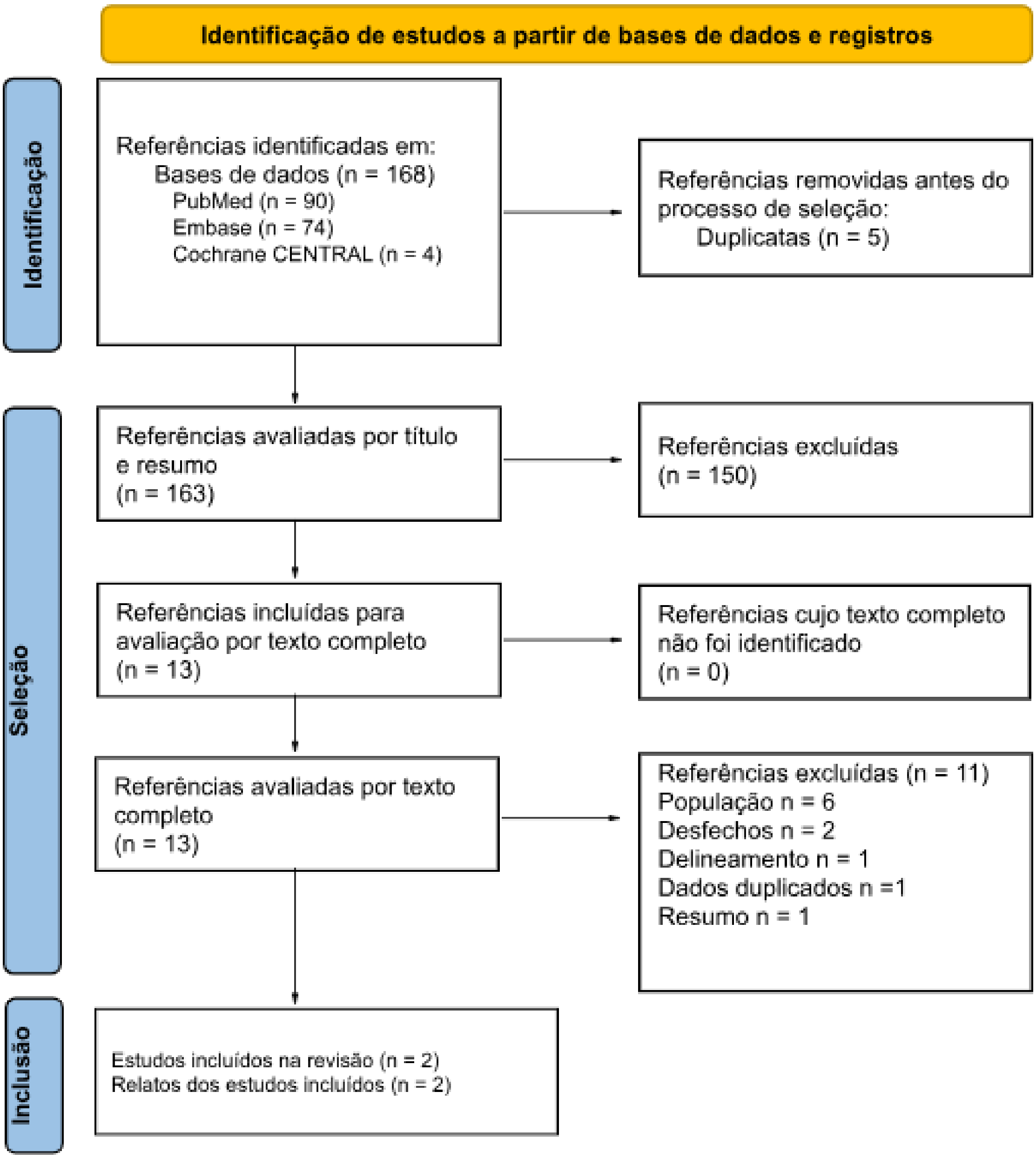

Fonte: elaboração própria

Tabela 1S. Características das revisões sistemáticas selecionadas.

Autor/ Ano	Tipo de estudos	Medicamento	População	Estudos primários	Motivo para não considerar atualização da revisão
Berbenetz and Mrkobrada, 2016	ECR	MRA	Pacientes com ICFER e ICFEP.	Akbulut, 2003; Boccanelli, 2009; Chan, 2007; Cicoira, 2002; Deswal, 2011; Edelmann, 2013; Edwards, 2009; Zannad, 2011; Pitt, 2003; Gao, 2007; Pitt, 2013; Pitt, 1999; Pitt, 2014; Udelson, 2010; Vizzardi, 2014.	Limitações metodológicas significativas avaliada pela ferramenta AMSTAR-2.<br><br>Os autores não registraram um protocolo prévio, nem justificaram a inclusão de ECRs. A busca foi limitada, e não houve lista de estudos excluídos ou análise de financiamento dos estudos incluídos. Além disso, realizaram a metanálise sem avaliar o impacto do risco de viés individual nos resultados, tampouco discutiram esse viés na interpretação dos achados.
Fukuta, 2018	ECR	MRA	Pacientes com ICFEP.	Mottram, 2004; Deswal, 2011; Edelmann, 2013; Kurrelmeyer, 2014; Kosmala, 2016; Upadhya, 2017.	Limitações metodológicas significativas avaliadas pela ferramenta AMSTAR-2.<br><br>Os autores não registraram um protocolo prévio, justificativa insuficiente para inclusão de ECRs e falta de lista de estudos excluídos. Os autores não relataram fontes de financiamento dos estudos primários nem avaliaram adequadamente o impacto do risco de viés nos resultados. Além disso, não explicaram satisfatoriamente

					a heterogeneidade observada, comprometendo a validade das conclusões.

Legenda: ECR, ensaio clínico randomizado; ICFER, insuficiência cardíaca com fração de ejeção reduzida, ICFEP, insuficiência cardíaca com fração de ejeção preservada, MRA, Antagonistas dos Receptores de Mineralocorticoides.

Referências: Berbenetz NM, Mrkobrada M. Mineralocorticoid receptor antagonists for heart failure: systematic review and meta-analysis. BMC Cardiovasc Disord. 2016 Dec 1;16(1):246. doi: 10.1186/s12872-016-0425-x. Fukuta H, Goto T, Wakami K, Kamiya T, Ohte N. Effects of mineralocorticoid receptor antagonists on left ventricular diastolic function, exercise capacity, and quality of life in heart failure with preserved ejection fraction: a meta-analysis of randomized controlled trials. Heart Vessels. 2019 Apr;34(4):597-606. doi: 10.1007/s00380-018-1279-1.

Fonte: elaboração própria.

Quadro 3S. Estratégia de busca bibliográfica para ensaios clínicos randomizados realizada em 02 de março de 2025

Base	Estratégia de busca	Registros recuperados
Medline	#1 "Mineralocorticoid Receptor Antagonists"[Mesh] OR "Mineralocorticoid Receptor Antagonists" OR (Antagonists, Mineralocorticoid Receptor) OR (Receptor Antagonists, Mineralocorticoid) OR (Mineralocorticoid Receptor Antagonist) OR (Antagonist, Mineralocorticoid Receptor) OR (Receptor Antagonist, Mineralocorticoid) OR (Aldosterone Receptor Antagonists) OR (Antagonists, Aldosterone Receptor) OR (Receptor Antagonists, Aldosterone) OR (Aldosterone Receptor Antagonist) OR (Antagonist, Aldosterone Receptor) OR (Receptor Antagonist, Aldosterone) OR (Mineralocorticoid Antagonist) OR (Antagonist, Mineralocorticoid) OR (Mineralocorticoid Antagonists) OR (Antagonists, Mineralocorticoid) OR (Aldosterone Antagonists) OR (Antagonists, Aldosterone) OR (Aldosterone Antagonist) OR (Antagonist, Aldosterone)<br><br>#2 "Mineralocorticoid Receptor Antagonists" [Pharmacological Action] OR (` drospirenone (Supplementary Concept)) OR (RU 28318 (Supplementary Concept)) OR (N-(3-(1-cyclopropyl-1-(2,4-difluorophenyl)ethyl)-1H-indol-7-yl)methanesulfonamide (Supplementary Concept)) OR (de-O-methyllasiodiplodin (Supplementary Concept)) OR (N-4,4-dimethyl-2-thioxo-1,4-dihydro-2H-3,1-benzoxazin-6-ylthiophene-2-sulfonamide (Supplementary Concept)) OR (6-(1-(4-fluoro-2-methylphenyl)-3-(trifluoromethyl)-1H-pyrazol-5-yl)-2H-1,4-benzoxazin-3(4H)-one (Supplementary Concept)) OR ((("Canrenoic Acid"[Mesh]) OR "Canrenone"[Mesh]) OR "Spironolactone"[Mesh]) OR "Eplerenone"[Mesh]	208

	#3 (Soludactone) OR (Soldactone) OR (Spiroctan) OR (Aldadiene) OR (Phanurane) OR (Verospirone) OR (Spirolactone) OR (Veroshpiron) OR (Aldactone) OR (Aldactone A) OR (Spirolang) OR (Aquareduct) OR (Duraspiron) OR (Espironolactona Alter) OR (Espironolactona Mundogen) OR (Flumach) OR (Frumikal) OR (Jenaspiron) OR (Novo-Spiroton) OR (NovoSpiroton) OR (Novo Spiroton) OR (Practon) OR (Spiractin) OR (Spiro L.U.T.) OR (Spiro Von Ct) OR (Ct, Spiro Von) OR (Von Ct, Spiro) OR (Spirobeta) OR (Spirogamma) OR (Spironone) OR (Spirospare) OR (Verospiron) OR (Spirono-Isis) OR (Spirono Isis) OR (Eplerenon) OR (Inspra)<br><br>#4 #1 OR #2 OR #3<br><br>#5 "Heart Failure, Diastolic"[Mesh] OR (Diastolic Heart Failures) OR (Diastolic Heart Failure) OR (Heart Failure, Preserved Ejection Fraction) OR (Heart Failure, Normal Ejection Fraction) OR (Preserved Ejection Fraction Heart Failure) OR (Normal Ejection Fraction Heart Failure)<br><br>#6 (randomized controlled trial[Publication Type] OR (randomized[Title/Abstract] AND controlled[Title/Abstract] AND trial[Title/Abstract]))<br><br>#4 AND #5 AND #6	
EMBASE	#1 'antimineralocorticoid'/exp OR 'antimineralocorticoid' OR 'mineralocorticoid receptor antagonists'/exp OR 'mineralocorticoid receptor antagonists' OR 'mineralocorticoid antagonist'/exp OR 'mineralocorticoid antagonist' OR 'aldosterone antagonism'/exp OR 'aldosterone antagonism' OR 'aldosterone antagonists'/exp OR 'aldosterone antagonists' OR 'anti aldosterone'/exp OR 'anti aldosterone' OR 'antialdosterone'/exp OR 'antialdosterone' OR 'antialdosterone	287

	agent'/exp OR 'antialdosterone agent' OR 'selective aldosterone receptor antagonist'/exp OR 'selective aldosterone receptor antagonist' OR 'aldosterone antagonist'/exp OR 'aldosterone antagonist'<br><br>#2 'diastolic dysfunction'/exp OR 'diastolic dysfunction' OR 'hf with preserved ejection fraction'/exp OR 'hf with preserved ejection fraction' OR 'hf-pef (heart failure with preserved ejection fraction)'/exp OR 'hf-pef (heart failure with preserved ejection fraction)' OR 'hfpef (heart failure with preserved ejection fraction)'/exp OR 'hfpef (heart failure with preserved ejection fraction)' OR 'heart failure with preserved ejection fraction'/exp OR 'heart failure with preserved ejection fraction'<br><br>#3 ('randomized controlled trial'/de OR 'controlled clinical trial'/de OR random*:ti,ab,tt OR 'randomization'/de OR 'intermethod comparison'/de OR placebo:ti,ab,tt OR compare:ti,tt OR compared:ti,tt OR comparison:ti,tt OR ((evaluated:ab OR evaluate:ab OR evaluating:ab OR assessed:ab OR assess:ab) AND (compare:ab OR compared:ab OR comparing:ab OR comparison:ab)) OR ((open NEXT/1 label):ti,ab,tt) OR (((double OR single OR doubly OR singly) NEXT/1 (blind OR blinded OR blindly)):ti,ab,tt) OR 'double blind procedure'/de OR ((parallel NEXT/1 group*):ti,ab,tt) OR crossover:ti,ab,tt OR 'cross over':ti,ab,tt OR (((assign* OR match OR matched OR allocation) NEAR/6 (alternate OR group OR groups OR intervention OR interventions OR patient OR patients OR subject OR subjects OR participant OR participants)):ti,ab,tt) OR assigned:ti,ab,tt OR allocated:ti,ab,tt OR ((controlled NEAR/8 (study OR design OR trial)):ti,ab,tt) OR volunteer:ti,ab,tt OR volunteers:ti,ab,tt OR 'human experiment'/de OR trial:ti,tt) NOT (((random* NEXT/1 sampl* NEAR/8 ('cross section*' OR questionnaire* OR survey OR surveys OR database OR	

	databases)):ti,ab,tt) NOT ('comparative study'/de OR 'controlled study'/de OR 'randomised controlled':ti,ab,tt OR 'randomized controlled':ti,ab,tt OR 'randomly assigned':ti,ab,tt) OR ('cross-sectional study' NOT ('randomized controlled trial'/de OR 'controlled clinical study'/de OR 'controlled study'/de OR 'randomised controlled':ti,ab,tt OR 'randomized controlled':ti,ab,tt OR 'control group':ti,ab,tt OR 'control groups':ti,ab,tt)) OR ('case control*':ti,ab,tt AND random*:ti,ab,tt NOT ('randomised controlled':ti,ab,tt OR 'randomized controlled':ti,ab,tt)) OR ('systematic review':ti,tt NOT (trial:ti,tt OR study:ti,tt)) OR (nonrandom*:ti,ab,tt NOT random*:ti,ab,tt) OR 'random field*':ti,ab,tt OR (('random cluster' NEAR/4 sampl*):ti,ab,tt) OR (review:ab AND review:it NOT trial:ti,tt) OR ('we searched':ab AND (review:ti,tt OR review:it)) OR 'update review':ab OR ((databases NEAR/5 searched):ab) OR ((rat:ti,tt OR rats:ti,tt OR mouse:ti,tt OR mice:ti,tt OR swine:ti,tt OR porcine:ti,tt OR murine:ti,tt OR sheep:ti,tt OR lambs:ti,tt OR pigs:ti,tt OR piglets:ti,tt OR rabbit:ti,tt OR rabbits:ti,tt OR cat:ti,tt OR cats:ti,tt OR dog:ti,tt OR dogs:ti,tt OR cattle:ti,tt OR bovine:ti,tt OR monkey:ti,tt OR monkeys:ti,tt OR trout:ti,tt OR marmoset*:ti,tt) AND 'animal experiment'/de) OR ('animal experiment'/de NOT ('human experiment'/de OR 'human'/de)))<br><br>#4 [embase]/lim NOT ([embase]/lim AND [medline]/lim)<br><br>#1 AND #2 AND #3 AND #4	
COCHRANE (CENTRAL)	#1 MeSH descriptor: [Mineralocorticoid Receptor Antagonists] explode all trees<br>#2 "Aldosterone Antagonist" OR "Aldosterone Antagonists" OR "Antagonists, Aldosterone" OR "Antagonist, Aldosterone" OR "Receptor Antagonists, Mineralocorticoid" OR " ntagonist, Aldosterone Receptor" OR "Receptor Antagonists, Aldosterone" OR "Aldosterone Receptor Antagonists" OR "Receptor	27

	Antagonist, Mineralocorticoid” OR “Antagonist, Mineralocorticoid Receptor” OR “Mineralocorticoid Receptor Antagonist” OR “Receptor Antagonist, Aldosterone” OR “Aldosterone Receptor Antagonist” OR “Mineralocorticoid Antagonist” OR “Antagonists, Mineralocorticoid” OR “Antagonist, Mineralocorticoid” OR “Mineralocorticoid Antagonists” OR “Antagonists, Aldosterone Receptor” OR “Antagonists, Mineralocorticoid Receptor” 1121<br>#3 MeSH descriptor: [Heart Failure, Diastolic] explode all trees<br>#4 “Heart Failure, Normal Ejection Fraction” OR “Diastolic Heart Failures” OR “Diastolic Heart Failure” OR “Heart Failure, Preserved Ejection Fraction” OR “Preserved Ejection Fraction Heart Failure”<br>#5 #1 OR #2<br>#6 #3 OR #4<br>#7 #5 AND #6	
Total		522

Fonte: elaboração própria

Figura 2S. Fluxograma PRISMA da busca por ensaios clínicos randomizados

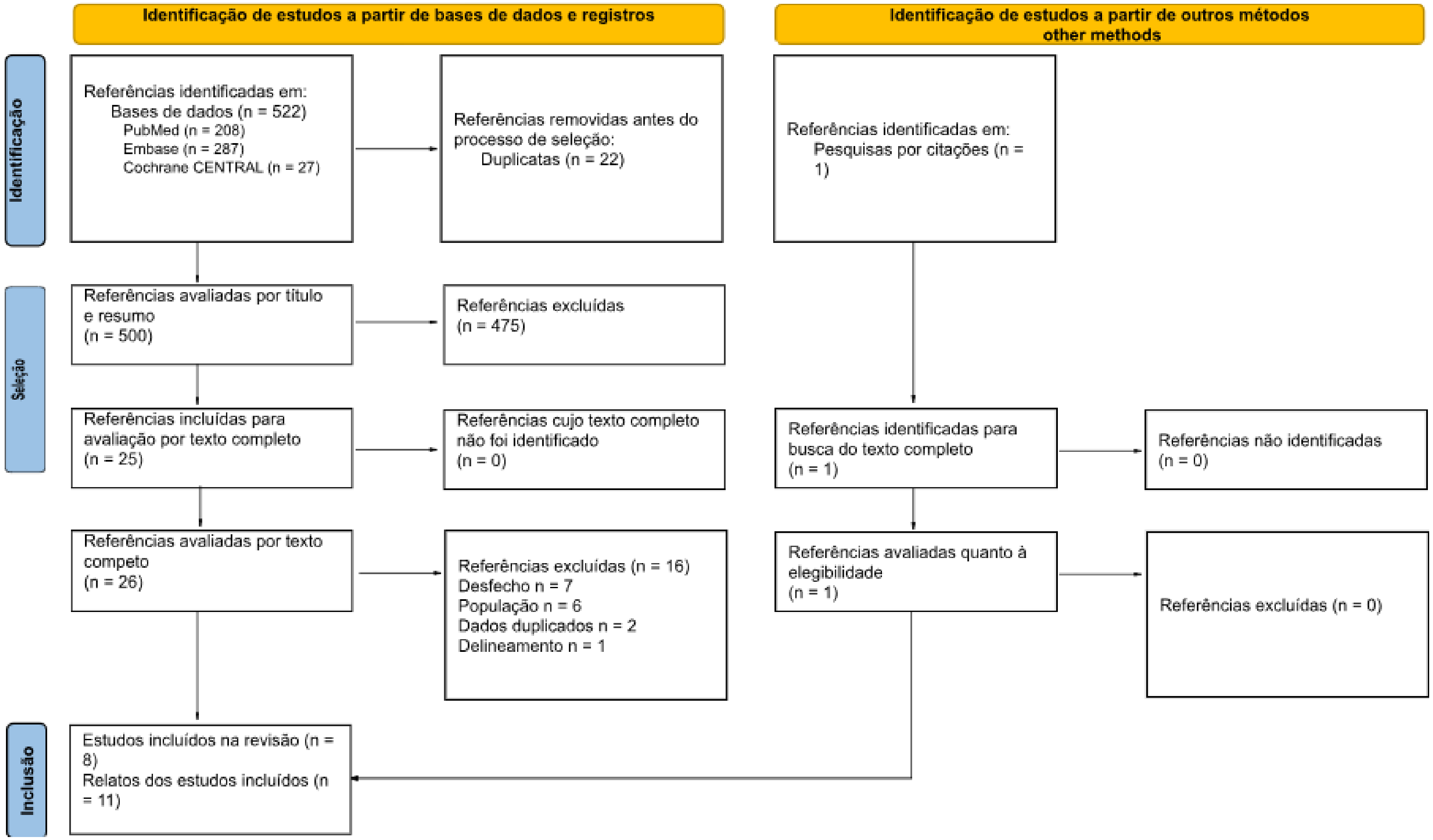

Fonte: elaboração própria

Quadro 3S. Estudos excluídos e motivos de exclusão

Autor / Ano	Título	Motivos para a exclusão
Rales Investigators, 1996	Effectiveness of spironolactone added to an angiotensin-converting enzyme inhibitor and a loop diuretic for severe chronic congestive heart failure (the Randomized Aldactone Evaluation Study [RALES])	População
Cicoira, 2004	Effects of ACE gene insertion/deletion polymorphism on response to spironolactone in patients with chronic heart failure	População
Cicoira, 2002	Long-term, dose-dependent effects of spironolactone on left ventricular function and exercise tolerance in patients with chronic heart failure	População
Desai, 2025	Finerenone in Patients With a Recent Worsening Heart Failure Event: The FINEARTS-HF Trial	Desfecho
Deswal, 2010	Randomized trial of aldosterone AntagonisM in diastolic heart failure (RAAM-DHF)	Dados duplicados
Holland, 2010	Extended duration of aldosterone blockade fails to improve diastolic dysfunction over the effects of blood pressure in patients with heart failure and normal ejection fraction	Desfecho
Karapysh, 2015	Effects of spironolactone on the left ventricular hypertrophy in chronic heart failure with preserved ejection fraction	Desfecho
Kasama, 2007	Additive effects of spironolactone and candesartan on cardiac sympathetic nerve activity and left ventricular remodeling in patients with congestive heart failure	População
Kosmala, 2016	Beneficial effect of aldosterone antagonism on exercise tolerance in heart failure with preserved ejection fraction-	Dados duplicados

	SpironolacTone in myocaRdial dysfunction with reduced exercise capacity (STRUCTURE)	
Kurgansky, 2019	Spironolactone reduces the risk of mortality and hospitalization in veterans with heart failure with preserved ejection fraction	Delineament o
Li, 2009	Treatment with spironolactone for 24 weeks decreases the level of matrix metalloproteinases and improves cardiac function in patients with chronic heart failure of ischemic etiology	População
McDiarmid, 2020	Myocardial Effects of Aldosterone Antagonism in Heart Failure With Preserved Ejection Fraction	Desfecho
Orea-Tejeda, 2007	Aldosterone receptor antagonists induce favorable cardiac remodeling in diastolic heart failure patients	Desfecho
Rousseau, 2002	Beneficial neurohormonal profile of spironolactone in severe congestive heart failure: results from the RALES neurohormonal substudy	População
Shah, 2014	Cardiac structure and function and prognosis in heart failure with preserved ejection fraction: findings from the echocardiographic study of the Treatment of Preserved Cardiac Function Heart Failure with an Aldosterone Antagonist (TOPCAT) Trial	Desfecho
Uzunhasan, 2009	Effects of aldosterone blockade on left ventricular function and clinical status during acute myocardial infarction	Desfecho

Fonte: elaboração própria.

Figura 3S. Avaliação da qualidade metodológica utilizando a ferramenta Risk of Bias 2.0.

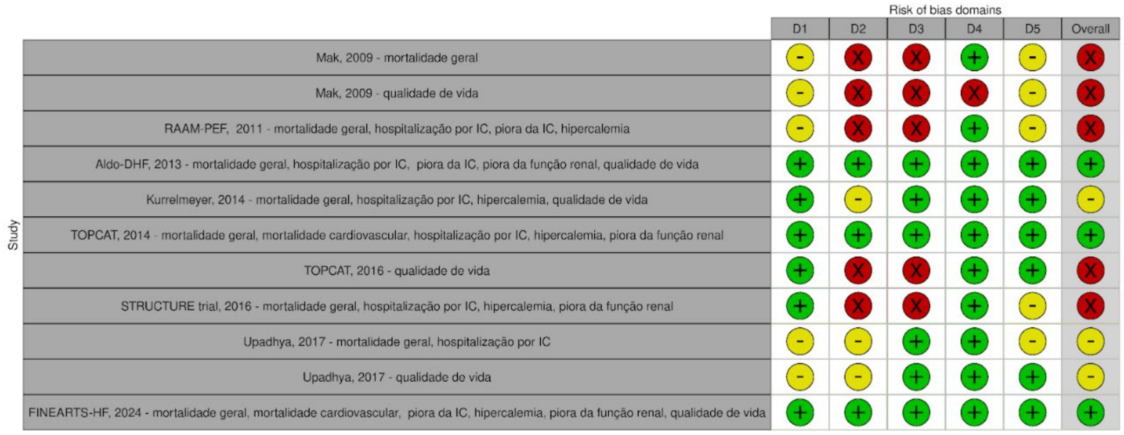

Círculos verdes representam baixo risco de viés, círculos amarelos representam algumas preocupações e círculos vermelhos representam alto risco de viés. D1: Viés no processo de randomização; D2: Viés devido a desvios da intervenção pretendida; D3: Viés devido a dados faltantes; D4: Viés na aferição dos desfechos; D5: Viés no relato dos desfechos.

Fonte: elaboração própria.

Tabela 2S. Tabela resumo de evidências para avaliação de eficácia e segurança dos antagonistas dos receptores de mineralocorticoides comparado ao controle para tratamento de pacientes com insuficiência cardíaca com fração de ejeção preservada

Avaliação da certeza da evidência							Sumário de Resultados				
Participantes (estudos) Seguimento	Risco de viés	Inconsistência	Evidência indireta	Imprecisão	Viés de publicação	Certeza da evidência	Taxas de eventos do estudo (%)		Efeito relativo (IC 95%)	Efeitos absolutos potenciais	
							Com controle	Com MRA		Risco com controle	Diferença de risco com Antagonistas dos Receptores de Mineralocorticoides
**Mortalidade geral (seguimento: variação 6 meses para 39,6 meses)**											
10234 (8 ECRs)	não grave[a]	não grave	não grave	grave[b]	nenhum	⊕⊕⊕◯ Moderada [a,b]	796/5110 (15.6%)	743/5124 (14.5%)	**RR 0.93** (0.85 para 1.02)	796/5110 (15.6%)	**11 menos por 1.000** (de 23 menos para 3 mais)
**Mortalidade cardiovascular (seguimento: variação 32 meses para 39,6 meses)**											
9446 (2 ECRs)	não grave	não grave	não grave	grave[b]	nenhum	⊕⊕⊕◯ Moderada [b]	402/4721 (8.5%)	436/4725 (9.2%)	**RR 0.92** (0.81 para 1.05)	402/4721 (8.5%)	**7 menos por 1.000** (de 16 menos para 4 mais)
**Hospitalização por IC (seguimento: variação 6 meses para 39,6 meses)**											

10040 (6 ECRs)	não grave[a]	não grave	não grave	não grave	nenhum	⊕⊕⊕⊕ Alta	781/5015 (15.6%)	678/5025 (13.5%)	**RR 0.87** (0.79 para 0.96)	781/5025 (15.6%)	**20 menos por 1.000** (de 33 menos para 6 mais)

Piora da insuficiência cardíaca

6467 (3 ECRs)	não grave	não grave	não grave	não grave	nenhum	⊕⊕⊕⊕ Alta	1105/3230 (34.2%)	912/3237 (28.2%)	**RR 0.82** (0.77 para 0.89)	1105/3230 (34.2%)	**62 menos por 1.000** (de 79 menos para 38 menos)

Qualidade de vida (seguimento: variação 9 meses para 12 meses; avaliado com: MLHFQ)

505 (3 ECRs)	não grave[c]	não grave	não grave	grave[b]	nenhum	⊕⊕◯◯ Baixa [b,c]	-	-	-	-	DM **1.17 menor** (3.1 menor para 0.76 mais alto)

Qualidade de vida (seguimento: variação 6 meses para 39,6 meses; avaliado com: KCCQ - Overall Summary Score)

7659 (3 ECRs)	não grave[d]	não grave	não grave	grave[b]	nenhum	⊕⊕◯◯ Baixa [b,d]	-	-	-	-	DM **3.62 mais alto** (1.86 menor para 9.1 mais alto)

Qualidade de vida (seguimento: média 6 meses; avaliado com: KCCQ - Clinical Summary Score)

92 (2 ECRs)	grave[e]	não grave	não grave	muito grave[f]	nenhum	⊕◯◯◯ Muito baixa [e,f]	-	-	-	-	DM **3.33 menor** (9.59 menor para 2.94 mais alto)

Hipercalemia (seguimento: variação 6 meses para 39,6 meses)

9654 (5 ECRs)	não grave[a]	não grave	não grave	não grave	nenhum	⨁⨁⨁⨁ Alta [a]	284/4830 (5.9%)	617/4824 (12.8%)	**RR 2.16** (1.89 para 2.17)	284/4830 (5.9%)	**68 mais por 1.000** (de 52 mais para 69 mais)
**Deterioração da função renal (seguimento: variação 6 meses para 32 meses)**											
6554 (3 ECRs)	não grave[a]	não grave	não grave	grave[b]	nenhum	⨁⨁⨁◯ Moderada [a,b]	59/3274 (1.8%)	86/3280 (2.6%)	**RR 1.53** (0.96 para 2.45)	59/3274 (1.8%)	**10 mais por 1.000** (de 1 menos para 26 mais)

DM: Diferença de médias; IC: Intervalo de confiança; RR: Razão de risco.

Explicações

a. Embora alguns estudos tenham apresentado risco de viés, os que mais influenciaram a análise foram classificados como de baixo risco de viés.

b. Intervalo de confiança amplo, abrangendo tanto a possibilidade de benefício quanto de risco, o que indica uma imprecisão nos resultados.

c. De acordo com a ferramenta RoB 2, o estudo Mak (2009) foi classificado com algumas preocupações, com penalizações nos domínios processo de randomização e relato seletivo dos desfechos. Já o estudo Upadhya (2017) foi avaliado como tendo algumas preocupações com penalizações nos domínios processo de randomização e desvios nas intervenções pretendidas.

d. De acordo com a ferramenta RoB 2, os estudos RAAM-PEF (2011) e TOPCAT (2016) foram classificados como de alto risco de viés, com penalizações em diversos domínios, comprometendo a confiabilidade da estimativa de efeito.

e. De acordo com a ferramenta RoB 2, o estudo RAAM-PEF (2011) foi classificado como de alto risco de viés, com penalizações em diversos domínios, comprometendo a confiabilidade da estimativa de efeito. Já o estudo Kurrelmeyer (2014) foi avaliado como tendo algumas preocupações no domínio desvios da intervenção pretendida.

f. Intervalo de confiança amplo, abrangendo tanto a possibilidade de benefício quanto de risco, o que indica uma imprecisão nos resultados. N pequeno.

Fonte: elaboração própria

Material suplementar 2

Das evidências para a recomendação

Para assegurar uma abordagem estruturada e transparente na avaliação das evidências e na formulação de recomendações, este parecer apresenta uma sugestão de *Evidence to Decision (EtD) Framework*, metodologia reconhecida por organizações como a OMS. O EtD permite a sistematização de critérios-chave para tomada de decisão, incluindo:

- Balanço entre benefícios e riscos – Análise comparativa dos impactos positivos e negativos da tecnologia/intervenção em questão.
- Certeza da evidência – Avaliação da qualidade e robustez dos estudos disponíveis (GRADE).
- Valores e preferências – Consideração das expectativas e prioridades dos stakeholders envolvidos.
- Custos da tecnologia/intervenção em questão.
- Viabilidade e aceitabilidade – Efeitos sobre viabilidade e barreiras de implementação.

O emprego do EtD visa garantir que a recomendação final seja não apenas tecnicamente embasada, mas também pragmaticamente aplicável, alinhada às necessidades da sociedade.

AVALIAÇÃO

Efeitos desejáveis (benefícios)<br>Qual a importância dos efeitos desejáveis previstos?
EVIDÊNCIAS DE PESQUISA
Impacto limitado em desfechos como mortalidade geral e mortalidade cardiovascular. MRA resulta na redução do risco de piora da insuficiência cardíaca e hospitalização por IC, contudo há variabilidade na definição do desfecho piora clínica.<br>Em relação à qualidade de vida, não foram identificadas diferenças estatisticamente significativas entre os grupos nas diversas escalas avaliadas (MLHFQ, KCCQ, EQ5D-VAS e SF-36).
Efeitos indesejados (riscos)<br>Qual a importância dos efeitos indesejáveis previstos?
EVIDÊNCIAS DE PESQUISA
Do ponto de vista da segurança, a terapia com MRA esteve associada a importantes eventos adversos, particularmente um aumento significativo no risco de hipercalemia e uma tendência à piora da função renal.
Certeza da evidência<br>Qual é o grau de certeza global da evidencia dos efeitos?
EVIDÊNCIAS DE PESQUISA
Certeza da evidência alta a moderada
Valores<br>Existe uma incerteza importante ou variabilidade na forma como as pessoas valorizam os principais resultados?
EVIDÊNCIAS DE PESQUISA
Pacientes podem priorizar a redução da progressão da doença (desfecho piora da IC), mesmo sem melhora na mortalidade ou qualidade de vida.<br>Falta de melhora na qualidade de vida: se o paciente não percebe benefícios concretos no dia a dia (ex.: capacidade de realizar atividades físicas), pode questionar a utilidade do medicamento.<br>Eventos adversos: O risco de hipercalemia (que pode ser assintomático, mas grave) e piora da função renal pode desencorajar a adesão, especialmente em idosos ou pacientes com comorbidades. Ainda, médicos podem ser cautelosos ao prescrever MRA devido aos riscos renais e metabólicos.

Viabilidade<br>É viável implementar a intervenção?
EVIDÊNCIAS DE PESQUISA
Tem efeito conhecido na melhora da função diastólica em pacientes com ICFEr e são amplamente disponíveis, o que facilita seu uso clínico.<br>Posologia: uso diário<br>Monitorização frequente: pode haver necessidade de exames regulares (dosagem de potássio e creatinina) pode ser vista como um incômodo ou barreira ao tratamento.

Critério de custo
EVIDÊNCIAS DE PESQUISA
1. Espironolactona 25 mg (30 comprimidos)<br>● Preço de fábrica (CMED PF 18%): R$ 34,12<br>● Dose usual para ICFEP: 25 mg/dia (1 comprimido/dia).<br>● Custo mensal: R$ 34,12.<br>2. Eplerenona 25 mg (30 comprimidos)<br>● Preço de fábrica (CMED PF 18%): R$ 41,87<br>● Dose usual para ICFEP: 25–50 mg/dia (1–2 comprimidos/dia).<br>○ Se 25 mg/dia: R$ 41,87/mês.<br>○ Se 50 mg/dia: R$ 83,74/mês.

**Sugestão de pontos a serem considerados para discussão e redação da recomendação.**
Considerações do subgrupo
● Preferência por algum dos MRA? (espironolactona e eplerenona)<br>● Pacientes com biomarcadores elevados (NT-proBNP alto) ou com sintomas persistentes, apesar da terapia padrão, seriam uma população que se beneficiaria?
Considerações sobre a implementação
● O monitoramento cuidadoso do potássio, da função renal e da dosagem de diuréticos no início e durante o acompanhamento é fundamental para minimizar o risco de hipercalemia e piora da função renal.<br>● Após o início de um MRA ou BRA (bloqueador do receptor da angiotensina), e com qualquer ajuste de dose, o potássio sérico e a creatinina devem ser monitorados na xx semana, na xx semana, no xx mês e sempre que clinicamente indicado.
