# Supplementary material for: Revisão Sistemática: Antagonistas dos Receptores de Mineralocorticoides na Insuficiência Cardíaca com Fração de Ejeção Preservada e Levemente Reduzida
Source: Arq Bras Cardiol. 2026 May 26;123(4):e20250622. [Article in Portuguese] doi: 10.36660/abc.20250622 (PMC13398873; doi:10.36660/abc.20250622)
Supplement: Translation

# Translation: Systematic Review: Mineralocorticoid Receptor Antagonists in Heart Failure with Preserved and Mildly Reduced Ejection Fraction

**DOI:** 10.36660/abc.20250622i

**Authors:** Marcus Vinicius Simões, Luiz Claudio Danzmann, Sílvia Marinho Martins, José Albuquerque de Figueiredo, Fabiana G. Marcondes-Braga, Ricardo Mourilhe-Rocha, Denilson Campos de Albuquerque, Mucio Tavares de Oliveira, Lidia Ana Zytynski Moura

**Affiliations:** 1 Brasil Universidade de São Paulo Faculdade de Medicina de Ribeirão Preto, Ribeirão Preto, SP – Brasil; 2 Brasil Universidade Luterana do Brasil, Canoas, RS – Brasil; 3 Brasil Hospital São Lucas da PUCRS, Porto Alegre, RS – Brasil; 4 Brasil Pronto Socorro Cardiológico de Pernambuco (PROCAPE), Recife, PE – Brasil; 5 Brasil Real Hospital Português de Beneficência em Pernambuco – Realcor, Recife, PE – Brasil; 6 Brasil Universidade Federal do Maranhão, São Luís, MA – Brasil; 7 Brasil Instituto do Coração do Hospital das Clínicas da Faculdade de Medicina da Universidade de São Paulo, São Paulo, SP – Brasil; 8 Brasil Universidade do Estado do Rio de Janeiro, Rio de Janeiro, RJ – Brasil; 9 Brasil Hospital Pró-Cardíaco – Américas Serviços Médicos, Rio de Janeiro, RJ – Brasil; 10 Brasil Pontifícia Universidade Católica do Paraná, Curitiba, PR – Brasil

**Keywords:** Mineralocorticoid Receptor Antagonists, Diastolic Heart Failure, Systolic Heart Failure, Systematic Review, Meta-Analysis

## Abstract

Heart failure with preserved ejection fraction (HFpEF) or mildly reduced ejection fraction (HFmrEF) is a prevalent condition associated with significant morbidity and mortality, and the benefits of mineralocorticoid receptor antagonists (MRAs) remain unclear. To develop a systematic review on the use of MRAs in patients with HFpEF or HFmrEF, a systematic review and meta-analysis were conducted using a broad search strategy in the following databases: MEDLINE, Embase, and Cochrane Central Register of Controlled Trials (CENTRAL). Randomized controlled trials that evaluated the use of MRAs in patients with HFpEF compared to control groups were included. The quality of evidence and the strength of the recommendation were assessed according to the Grading of Recommendations Assessment, Development, and Evaluation (GRADE) methodology. Eight studies were included. No statistically significant differences were observed for the outcomes of overall mortality and cardiovascular mortality compared to the control group for any of the MRAs. A benefit was observed with a reduced risk of hospitalization for heart failure (relative risk: 0.87; 95% confidence interval: 0.79 to 0.96; moderate certainty of evidence) and of worsening heart failure events (relative risk: 0.83; 95% confidence interval: 0.77 to 0.89; high certainty of evidence). The results of the meta-analysis indicate a significant benefit from MRAs, spironolactone or finerenone, to reduce hospitalizations and worsening heart failure events in patients with HFpEF or HFmrEF. Mineralocorticoid Receptor Antagonists; Diastolic Heart Failure; Systolic Heart Failure; Systematic Review; Meta-Analysis.

## Introduction

In patients with heart failure with reduced ejection fraction, the use of steroidal mineralocorticoid receptor antagonists (MRAs), namely spironolactone and eplerenone, has clearly demonstrated a reduction in the risk of death and hospitalization. Evidence for this effect is derived from two pivotal clinical trials, the Randomized Aldactone Evaluation Study (RALES)^1^ and the Eplerenone in Mild Patients Hospitalization and Survival Study in Heart Failure (EMPHASIS-HF).^2^ Accordingly, international guidelines put forth strong and consistent recommendations for the use of these MRAs in patients with heart failure with reduced ejection fraction.^3^

In contrast, the efficacy of these medications for treating heart failure with mildly reduced ejection fraction (HFmrEF) or preserved ejection fraction (HFpEF) remains uncertain. Until recently, a single multicenter randomized clinical trial (RCT), Treatment of Preserved Cardiac Function Heart Failure with an Aldosterone Antagonist (TOPCAT),^4^ had tested the effect of spironolactone in patients with left ventricular ejection fraction (LVEF) > 45%. In this study, spironolactone did not significantly reduce the primary composite endpoint of first hospitalization for heart failure, resuscitated cardiac arrest, or cardiovascular death. However, analysis of the components of the primary outcome showed a reduction in hospitalizations for heart failure, indicating a potentially significant effect of spironolactone.^4^ Additionally, subsequent analyses suggested explanations for the study’s neutral result, observing that a considerable portion of the patients enrolled at sites in Russia and Georgia may not have had heart failure, given that the rate of observed events was much lower than that seen in patients enrolled in 4 countries in North and South America (United States, Canada, Brazil, and Argentina).^5^ Another post-hoc analysis indicated that many patients may not have taken the assigned medication, as no metabolite of the drug was detected in the urine of many patients randomized to spironolactone, indicating a possible benefit in patients who actually used it.^6^ As a consequence of these controversial aspects, recommendations for the use of MRAs in HFmrEF and HFpEF are weak or nonexistent.^7^

Recently, the Finerenone Trial to Investigate Efficacy and Safety Superior to Placebo in Patients With Heart Failure (FINEARTS-HF) evaluated the efficacy of finerenone, a non-steroidal MRA, in patients with LVEF > 40%, demonstrating a significant reduction in the risk of the primary composite outcome of worsening heart failure and cardiovascular death in patients with HFpEF and HFmrEF.^8^

Given the growing evidence suggesting potential benefits of MRAs in patients with heart failure with non-reduced ejection fraction, a systematic review was conducted with the objective of identifying, assessing, and summarizing the available scientific evidence regarding the efficacy and safety of these drugs in patients with HFpEF and HFmrEF.

## Methods

In order to develop this systematic review from the Brazilian Society of Cardiology (SBC), a rapid systematic review was conducted, following the Cochrane criteria.^9^ The review protocol was registered on the Open Science Framework platform, under number 10.17605/OSF.IO/MVB75. This study is reported in accordance with the Preferred Reporting Items for Systematic Reviews and Meta-Analyses (PRISMA) guidelines.^10^

The research question, structured in PICO format, sought to evaluate the efficacy and safety of the use of MRAs in patients with HFpEF and HFmrEF when compared to usual treatment or optimized medical therapy.

The inclusion criteria were as follows: (1) RCTs; (2) patients diagnosed with HFpEF (LVEF ≥ 40%); (3) symptomatic patients classified in New York Heart Association functional class II, III, or IV; (4) studies evaluating the use of MRAs as an intervention and including a control group using placebo or standard treatment for HFpEF.

The following exclusion criteria were applied: (1) patients with an estimated glomerular filtration rate < 25 mL/min/1.73 m^2^; (2) patients with serum potassium levels > 5.0 mmol/L; (3) studies that did not report an adequate comparator group (placebo or standard treatment); (4) narrative reviews, case reports, editorials, and preclinical studies (in animals or in vitro).

The primary efficacy outcomes included overall mortality, cardiovascular mortality, hospitalization for heart failure, worsening heart failure, and quality of life. The safety outcomes analyzed were hyperkalemia and worsening renal function.

Initially, a search for systematic reviews on the topic was conducted on February 26, 2025, in the following 3 databases: Embase, MEDLINE (via PubMed), and Cochrane Library. Details on the methodology and search strategies are available in the supplementary material (Chart 1S). However, due to the absence of methodologically high-quality systematic reviews to support the recommendations, a new search for RCTs was carried out in the same databases, including a complementary manual search, based on the systematic reviews selected in the first stage. The supplementary material (Table 2S and Figure S1) display details about the search for systematic reviews.

The search for RCTs was restricted to studies published in English or Portuguese. Details on the search strategy are described in the supplementary material (Table 3S). Study selection was carried out using the Rayyan^11^ reference manager and evaluated by a single reviewer. The same reviewer conducted full-text screening. Data extraction was carried out by one reviewer using a previously tested form and validated by a second reviewer.

### Statistical analysis

Data were synthesized in narrative form; when possible, a meta-analysis was conducted for each outcome of interest, summarizing the results obtained in the RCTs.

Meta-analyses were performed using a random-effects model, with data from the included RCTs. For dichotomous outcomes, the relative risk (RR) was used as a measure to estimate the effect size. For continuous outcomes, the mean difference (MD) was used. A 95% confidence interval (CI) was considered. Heterogeneity was assessed using the I^2^ value, with values above 50% indicating significant heterogeneity. Meta-analyses were conducted using R software, version 4.4.3, with the meta package, version 8.0-2.^12^

Subgroup analyses were planned for the primary outcomes, provided data were available from the included studies. Sensitivity analyses for the primary outcomes were also planned, in the event that there were a sufficient number of methodologically homogeneous studies, excluding those that presented at least one domain assessed as having a high risk or some concerns of bias. However, these analyses were not necessary, given that the included studies showed low methodological and outcome heterogeneity. Publication bias assessment would have been performed through visual inspection of funnel plots if at least 10 studies had been grouped in a meta-analysis.

### Risk of bias assessment and certainty of evidence

Eligible systematic reviews were assessed for methodological quality using the AMSTAR-2 tool.^13^ The risk of bias of individual RCTs was assessed using the Cochrane Collaboration Risk of Bias 2.0 (RoB 2.0) tool.^14^ The overall certainty of evidence and the strength of recommendation were analyzed according to the approach developed by the Grading of Recommendations Assessment, Development, and Evaluation (GRADE) Working Group.^15^

## Results

### Recommendation

The results of the meta-analysis show that there was no statistically significant difference in overall and cardiovascular mortality outcomes, with moderate certainty of evidence. Regarding quality of life, the certainty of evidence was low, with no statistically significant difference. On the other hand, there was a significant increase in the incidence of hyperkalemia, with high certainty of evidence. Nonetheless, when considering the body of evidence, the panel assigned greater weight to the benefits observed in the clinical outcomes considered most relevant to patients, such as reduced hospitalizations for heart failure and clinical worsening, judging that these benefits outweigh the identified risk of hyperkalemia. However, it is worth emphasizing that patients’ values and preferences should be included in the decision-making process.

### Summary of evidence

Initially, a search for systematic reviews was conducted, resulting in the identification of 168 references in the databases. After removing 5 duplicate records, 163 references were analyzed by title and abstract, and 13 were selected for full reading. At the end of this stage, 2 references were included,^16,17^ as shown in Figure 1S.

The assessment of the risk of bias of these two systematic reviews, using the AMSTAR-2 tool, revealed significant methodological limitations in both studies (Table 1S). Therefore, a decision was reached to conduct a new search for RCTs that evaluated the efficacy and safety of MRAs in patients with HFpEF.

The search for RCTs identified 522 records. After excluding 22 duplicate records and 475 studies based on title and abstract, 27 references were selected for full-text reading. Finally, 8 studies reported in 11 publications were included in the systematic review and meta-analysis (Figure 2S). The lists of excluded studies and their respective justifications are detailed in the supplementary material (Chart 3S).

Of the 8 RCTs included, 5 compared spironolactone versus placebo, 2 eplerenone versus placebo, and 1 finerenone versus placebo.^4,5,8,18-26^ The follow-up time of the studies ranged from 6 to 60 months.

Table 1 displays the characteristics of the included studies whose results were identified.

**Table 1:** – Characteristics of the randomized clinical trials included (n = 8 trials, 11 references)

Study (author, year)	Mak, 2009^22^	RAAM-PEF (Deswal, 2011)^24^	Aldo-DHF (Edelmann, 2013)^18^	Kurrelmeyer, 2014^20^	TOPCAT (Pitt, 2014, Pfeffer, 2015 e Lewis, 2016)^4,5,21^	STRUCTURE trial (Kosmala, 2016)^19^	Upadhya, 2017^23^	FINEARTS-HF (Solomon, 2024 e Cunningham, 2025)^8,25^
**Study design and protocol registry**	Parallel-group, open RCT NCT00505336	Phase IV, parallel-group, double-blind RCT NCT00108251	Phase IIb, arallel-group, double-blind RCT ISRCTN94726526	Phase IV, parallel-group, double-blind RCT NCT00206232	Phase III, parallel-group, double-blind RCT NCT00094302	Phase IV, parallel-group, double-blind RCT ACTRN12614000088640	Phase III, parallel-group, double-blind RCT NCT00123955	Phase III, parallel-group, double-blind RCT NCT04435626
**Location and period**	Ireland Beginning April 2006	United States Beginning August 2006	Germany and Austria (10 sites) Beginning March 2007	United States Beginning July 2004	United States, Canada, Brazil, Argentina, Russia, and Georgia Beginning August 2006	Poland Beginning November 2011	United States Beginning April 2005	Argentina, Australia, Austria, Brazil, Bulgaria, Canada, China, Colombia, Czech Republic, Denmark, Finland, Germany, Greece, Hong Kong, Hungary, India, Israel, Italy, Japan, Korea, Latvia, Lithuania, Malaysia, Mexico, Netherlands, New Zealand, Poland, Portugal, Romania, Russia, Slovakia, Spain, Taiwan, Turkey, Ukraine, United Kingdom, United States Beginning September 2020
**Participants**	Individuals of both sexes with heart failure; ejection fraction ≥ 45% (n = 44) Age (mean): 80 ± 7.8 years Comorbidities: hypertension (91%); diabetes mellitus (28%); hyperlipidemia (29%)	Individuals of both sexes with heart failure; ejection fraction ≥ 50% (n = 46) Age (mean): 70 ± 9 years Comorbidities: hypertension (100%); diabetes mellitus (61%); CAD (57%); atrial fibrillation (13.7%)	Individuals of both sexes with heart failure; ejection fraction ≥ 50% (n = 422) Age (mean): 67 ± 8 years Comorbidities: hypertension (92%); hyperlipidemia (65%); CAD (40%); diabetes mellitus (17%); atrial fibrillation (5%); COPD (3%)	Individuals of both sexes with heart failure; ejection fraction ≥ 50% (n = 48) Age (mean): 71 ± 5 years Comorbidities: hypertension (83%); diabetes mellitus (38%); CAD (35%); atrial fibrillation (25%)	Individuals of both sexes with heart failure; ejection fraction ≥ 45% (n = 3,445) Age (median): 68.7 years Comorbidities: hypertension (91.5%); dyslipidemia (60.2%); CAD (58.8%); chronic kidney disease (38.7%); diabetes mellitus (32.5%); atrial fibrillation (35.3%); myocardial infarction (26%); COPD (11.7%); stroke (7.7%)	Individuals of both sexes with heart failure; ejection fraction ≥ 50% (n = 150) Age (mean): 67 ± 8 years Comorbidities: hypertension (91.5%); diabetes mellitus (39.5%)	Individuals of both sexes with heart failure; ejection fraction ≥ 50% (n = 80) Age (mean): 71 ± 1 years Comorbidities: hypertension (87.5%); diabetes mellitus (23%); pulmonary edema (19%)	Individuals of both sexes with heart failure; ejection fraction ≥ 40% (n = 7,463) Age (mean): 72 ± 9.7 years Comorbidities: hypertension (88.8%); diabetes mellitus (40.7%); atrial fibrillation (38.2%)
**Intervention**	Eplerenone 25 mg daily for 6 months, increased to 50 mg daily from 6 to 12 months (n = 24) 12 months	Eplerenone 25 mg daily for 2 weeks, increased to 50 mg for 22 weeks (n = 23) 6 months	Spironolactone 25 mg daily (n = 213) 12 months	Spironolactone 25 mg daily (n = 24) 6 months	Spironolactone 15 mg once daily, increased to a maximum of 45 mg daily during the first 4 months (n = 1,722) 39.6 months and 60 months	Spironolactone 25 mg daily (n = 75) 6 months	Spironolactone 25 mg daily (n = 42) 9 months	Finerenone at a maximum dose of 20 mg or 40 mg once daily (n = 3,003) 32 months
**Comparator**	Control group without additional treatment (n = 20) 12 months	Placebo (n = 23) 6 months	Placebo (n = 209) 12 months	Placebo (n = 24) 6 months	Placebo (n = 1,723) 39.6 months and 60 months	Placebo (n = 75) 6 months	Placebo (n = 38) 9 months	Placebo (n = 2,998) 32 months
**Outcomes and time points assessed**	Quality of life, biomarkers, diastolic function 12 months	Functional capacity, quality of life, biomarkers, diastolic function, functional class 6.4 months	Capacidade funcional, função diastólica, mortalidade geral, hospitalização, qualidade de vida, ansiedade, depressão, Events adversos 12 meses	Functional capacity, diastolic function, biomarkers, overall mortality, hospitalization for heart failure, hyperkalemia, and quality of life 6 months	Overall mortality, cardiovascular mortality, hospitalization, adverse events, hyperkalemia, and worsening renal function 39.6 months Quality of life 60 months	Functional capacity, diastolic function, overall mortality, hospitalization, biomarkers, adverse events 6 months	Functional capacity, diastolic function, mortality, hospitalization, adverse events, adherence, quality of life 9 months	Mortality, hospitalization, worsening heart failure, quality of life, adverse events, functional class 32 months
**Funding sources**	St Vincent’s University Hospital, Ireland	Pfizer Pharmaceuticals	German-Austrian Heart Failure Study Group and German Competence Network of Heart Failure	Women’s Fund; Houston, Texas	National Heart, Lung, and Blood Institute, National Institutes of Health (HHSN268200425207C)	Wroclaw Medical University (ST-678) e Royal Hobart Hospital Foundation (13-024)	National Institutes of Health (NIH, R01AG18915); Claude D. Pepper Older Americans Independence Center of Wake Forest University (P30AG21332); the Clinical and Translational Science Institute of Wake Forest School of Medicine (NIH UL1TR001420); e o Kermit G. Phillips Chair in Cardiovascular Medicine of Wake Forest School of Medicine	Bayer

CAD: coronary artery disease; COPD: chronic obstructive pulmonary disease; RCT: randomized clinical trial.

### Overall mortality

The use of MRAs did not result in a significant difference in the outcome of overall mortality when compared to the control group (RR: 0.93; 95% CI: 0.85 to 1.02; n = 10,215; 8 RCTs; I^2^ = 0%; moderate certainty of evidence; Figure 1 and Table 2S). The certainty of evidence was considered moderate due to imprecision of the data, with the CI including both a reduction of up to 15% and an increase of 2% in the risk of overall mortality.

**Figure 1:** – Effect of mineralocorticoid receptor antagonists versus control for the outcome of overall mortality. CI: confidence interval; MRAs: mineralocorticoid receptor antagonists; RR: relative risk. Source: the authors.

A subanalysis was performed by replacing the TOPCAT (2014) study with data from the subpopulation enrolled in the Americas, as described by Pfeffer et al. (TOPCAT 2015),^5^ due to the significant heterogeneity among the regions participating in the original study. The decision was based on the low event rate observed in the Russia/Georgia cohort, which compromises the external validity of the overall results. The clinical characteristics and response to intervention in the Americas subpopulation were more compatible with the other studies included in the meta-analysis. With this substitution, the use of MRAs continued to show no significant difference for the outcome of overall mortality when compared to the control group (RR: 0.91; 95% CI: 0.83 to 1.00; n = 8,537; 8 RCTs; I^2^ = 0%; Figure 2), maintaining moderate certainty of evidence.

**Figure 2:** – Subanalysis of the effect of mineralocorticoid receptor antagonists versus control for the outcome of overall mortality. CI: confidence interval; MRAs: mineralocorticoid receptor antagonists; RR: relative risk. Source: the authors.

### Cardiovascular mortality

The use of MRAs did not result in a significant difference in the outcome of cardiovascular mortality when compared to the control group (RR: 0.92; 95% CI: 0.81 to 1.05; n = 9,446; 2 RCTs; I^2^ = 0%; moderate certainty of evidence; Figure 3 and Table 2S). The certainty of evidence was considered moderate, with a wide CI, encompassing a 19% reduction to a 5% increase in risk, reflecting imprecision in the findings.

**Figure 3:** – Effect of mineralocorticoid receptor antagonists versus control for the outcome of cardiovascular mortality. CI: confidence interval; MRAs: mineralocorticoid receptor antagonists; RR: relative risk. Source: the authors.

In the subanalysis replacing the TOPCAT (2014) study with data from the subpopulation enrolled in the Americas, as described by Pfeffer et al. (TOPCAT 2015),^5^ the use of MRAs continued to show no significant difference in the outcome of cardiovascular mortality compared to the control group (RR: 0.85; 95% CI: 0.70 to 1.05; n = 7,768; 2 RCTs; I^2^ = 48.1%; Figure 4), maintaining moderate certainty of evidence.

**Figure 4:** – Subanalysis of the effect of mineralocorticoid receptor antagonists versus control for the outcome of cardiovascular mortality. CI: confidence interval; MRAs: mineralocorticoid receptor antagonists; RR: relative risk. Source: the authors.

### Hospitalization

The use of MRAs resulted in a difference in the outcome of cardiovascular hospitalization when compared to the control group (RR: 0.87; 95% CI: 0.79 to 0.96; n = 10,040; 6 RCTs; I^2^ = 18.4%; moderate certainty of evidence; Figure 5 and Table 2S). The certainty of evidence was considered moderate, with a wide 95% CI, encompassing a 4% to 21% reduction in risk.

**Figure 5:** – Effect of mineralocorticoid receptor antagonists versus control for the outcome of hospitalization for heart failure. CI: confidence interval; MRAs: mineralocorticoid receptor antagonists; RR: relative risk. Source: the authors. The data from FINEARTS-HF refer to the effect of finerenone compared to placebo for initial worsening heart failure events.21

The STRUCTURE study evaluated 64 patients in the intervention group and 67 patients in the control group. After 6 months of treatment, 3 hospitalizations for cardiac causes were recorded in the MRA-treated group (4.7%) and 4 hospitalizations in the control group (6.0%). However, the authors did not detail the diagnostic or clinical criteria used to define hospitalization for cardiac causes; therefore, the study was not included in this meta-analysis.

In the subanalysis replacing the TOPCAT (2014) study with data from the subpopulation enrolled in the Americas, as described by Pfeffer et al. (TOPCAT 2015),^5^ the use of MRAs continued to show the same significant difference for the outcome of cardiovascular hospitalization compared to the control group (RR: 0.87; 95% CI: 0.79 to 0.96; n = 8,362; 6 RCTs; I^2^ = 17.4%; Figure 6), maintaining moderate certainty of evidence.

**Figure 6:** – Subanalysis of the effect of mineralocorticoid receptor antagonists versus control for the outcome of cardiovascular hospitalization. CI: confidence interval; MRAs: mineralocorticoid receptor antagonists; RR: relative risk. Source: the authors. The data from FINEARTS-HF refer to the effect of finerenone compared to placebo for initial worsening heart failure events.21

### Worsening heart failure

The use of MRAs resulted in an 18% reduction in the risk of worsening heart failure when compared to the control group (RR: 0.82; 95% CI: 0.77 to 0.89; n = 6,467; 3 RCTs; I^2^ = 0%; high certainty of evidence; Figure 7 and Table 2S). A sensitivity analysis was performed excluding the Aldo-DHF study, as the population had relatively stable heart failure (without elevated NT-proBNP). In this analysis, the use of MRAs maintained a significant risk reduction (RR: 0.82; 95% CI: 0.76 to 0.88; 2 RCTs; n = 6,045), reinforcing the robustness of the findings.

**Figure 7:** – Effect of mineralocorticoid receptor antagonists versus control for the outcome of worsening heart failure. CI: confidence interval; MRAs: mineralocorticoid receptor antagonists; RR: relative risk. Source: the authors.

The included studies adopted varying definitions to characterize worsening heart failure. The RAAM-PEF study (2011) defined worsening as hospitalization for heart failure or the need for intensified treatment, including aMDinistration of intravenous diuretics or an increase in the dose of oral diuretics. The Aldo-DHF study (2013) considered worsening criteria as intensifying dyspnea, as well as worsening or onset of edema. The FINEARTS-HF study (2024) defined worsening heart failure as the occurrence of unplanned hospitalization, whether initial or recurrent, or an urgent visit due to the condition.

### Quality of life

The outcome of quality of life was assessed using the Minnesota Living With Heart Failure Questionnaire (MLHFQ), Kansas City Cardiomyopathy Questionnaire (KCCQ), EQ5D Visual Analog Scale (VAS), and 36-Item Short Form Health Survey (SF-36).

In the analysis of studies that used the MLHFQ, the use of MRAs did not result in a significant difference in outcome of quality of life compared to the control group (MD: −1.17; 95% CI: −3.10 to 0.76; n = 505; 3 RCTs; I^2^ = 0%; moderate certainty of evidence; Figure 8 and Table 2S). The certainty of evidence was considered moderate due to imprecision of the data, with a CI ranging from a possible mild improvement (3.1 points) to a minimal worsening (0.76 points). Additionally, the 2017 study by Upadhya et al.^23^ assessed the emotional and physical domains of the MLHFQ, and no significant differences were observed between the spironolactone and placebo groups after 6 months of treatment.

**Figure 8:** – Mean difference between mineralocorticoid receptor antagonists and control for the outcome of quality of life assessed by the MLHFQ. CI: confidence interval; MD: mean difference; MRAs: mineralocorticoid receptor antagonists; SD: standard deviation. Source: the authors.

Among the studies that used the KCCQ Overall Summary Score, no significant difference was observed between the groups (MD: 3.62; 95% CI: −1.85 to 9.10; n = 7,659; 3 RCTs; I^2^ = 92%; moderate certainty of evidence; Figure 9 and Table 2S). The certainty of the evidence was considered moderate due to imprecision, with a wide CI ranging from a slight worsening (−1.85 points) to a modest improvement (9.10 points). Similar results were observed for the Clinical Summary Score (MD: −3.33; 95% CI: −9.59 to 2.94; 2 RCTs, n = 92; I^2^ = 0%; Figure 10 and Table 2S), with low certainty, however, due to the small sample size and imprecision of the results, where the CI ranged from a possible worsening (−9.59) to a benefit (2.94) (Table 2S).

**Figure 9:** – Mean difference between mineralocorticoid receptor antagonists and control for the outcome of quality of life assessed by the KCCQ Overall Summary Score. CI: confidence interval; MD: mean difference; MRAs: mineralocorticoid receptor antagonists; SD: standard deviation. Source: the authors.

**Figure 10:** – Mean difference between mineralocorticoid receptor antagonists and control for the outcome of quality of life assessed by the KCCQ Clinical Summary Score. CI: confidence interval; MD: mean difference; MRAs: mineralocorticoid receptor antagonists; SD: standard deviation. Source: the authors.

The 2016 TOPCAT study also assessed quality of life using the EQ5D VAS. No statistically significant difference was observed in the mean score variation between the groups treated with spironolactone (mean variation: 9.04; standard error: 0.96) and placebo (mean variation: 8.67; standard error: 0.86) over 60 months of follow-up.

In the 2013 Aldo-DHF study, the use of spironolactone did not result in a significant improvement in the quality of life outcome assessed by the SF-36 Physical Functioning (adjusted difference of 1 point; 95% CI: −2 to 4; p = 0.62) and SF-36 Global Self-Assessment (adjusted difference of 0.0; 95% CI: −0.1 to 0.1; p = 0.79) when compared to the placebo group after 12 months of treatment.

### Hyperkalemia

The results demonstrated that the use of MRAs increased the risk of hyperkalemia compared to the control group (RR: 2.16; 95% CI: 1.89 to 2.47; n = 9,654; 5 RCTs; I^2^ = 0%; high certainty of evidence; Figure 11 and Table 2S). The CI indicated that MRA treatment increased the risk of hyperkalemia, with an estimated increase ranging from 89% to 147%.

**Figure 11:** – Effect of mineralocorticoid receptor antagonists versus control for the outcome of hyperkalemia. CI: confidence interval; MRAs: mineralocorticoid receptor antagonists; RR: relative risk. Source: the authors.

In the subanalysis replacing the TOPCAT (2014) study with data from the subpopulation enrolled in the Americas, as described by Pfeffer et al. (TOPCAT 2015),^5^ the use of MRAs continued to show an increased risk of hyperkalemia compared to the control group (RR: 2.54; 95% CI: 2.09 to 3.08; n = 7,976; 5 RCTs; I^2^ = 0%), maintaining moderate certainty of evidence (Figure 12).

**Figure 12:** – Subanalysis of the effect of mineralocorticoid receptor antagonists versus control for the outcome of hyperkalemia. CI: confidence interval; MRAs: mineralocorticoid receptor antagonists; RR: relative risk. Source: the authors.

### Worsening renal function

Analysis revealed that the use of MRAs showed a trend towards worsening renal function compared to the control (RR: 1.53; 95% CI: 0.96 to 2.45; n = 6,554; 3 RCTs; I^2^ = 0%; moderate certainty of evidence; Figure 13 and Table 2S). The certainty of evidence was considered moderate due to imprecision of the data; although the RR suggests a potential increase of 53%, the wide CI encompasses a marginal reduction of 4% to a significant increase of 145%.

**Figure 13:** – Effect of mineralocorticoid receptor antagonists versus control for the outcome of worsening renal function. CI: confidence interval; MRAs: mineralocorticoid receptor antagonists; RR: relative risk. Source: the authors.

### Risk of bias and certainty of evidence

The risk of bias of the RCTs was assessed across all outcomes. The Aldo-DHF, TOPCAT (2014), and FINEARTS-HF (2024) studies did not show bias in any of the domains assessed, corresponding to 34% of the included studies. Of the 9 publications included, 4 (44%) were classified as having a high risk of bias, whereas 2 studies (22%) had some concerns regarding the risk of bias in their overall assessment. Figure 3S displays a chart with the details of the judgments made for each domain.

The certainty in the final body of evidence was assessed using the GRADE approach for all outcomes of comparison, considering only the longest follow-up time points and the overall sample (Table 2 and Table 2S).

**Table 2:** – Assessment of the certainty of the body of evidence

Population: Patients with heart failure with preserved ejection fraction Intervention: MRAs Comparator: Control						
Outcomes	Potential absolute effects (95% CI)		Relative effect (95% CI)	Number of participants (studies)	Certainty of evidence (GRADE)	Narrative statements
	Risk with control	Risk with MRAs				
**Overall mortality** Follow-up: varying from 6 months to 39.6 months	156 per 1,000	**145 per 1,000** (132 to 159)	**RR 0.93** (0.85 to 1.02)	10,234 (8 RCTs)	⨁⨁⨁◯ Moderate^a,b^	MRAs probably do not result in a difference in the risk of overall mortality.
**Cardiovascular mortality** Follow-up: varying from 32 months to 39.6 months	85 per 1,000	**78 per 1,000** (69 to 89)	**RR 0.92** (0.81 to 1.05)	9,446 (2 RCTs)	⨁⨁⨁◯ Moderate^b^	MRAs probably do not result in a difference in the risk of cardiovascular mortality.
**Hospitalization for heart failure** Follow-up: varying from 6 months to 39.6 months	156 per 1,000	**135 per 1,000** (123 to 150)	**RR 0.87** (0.79 to 0.96)	10,040 (6 RCTs)	⨁⨁⨁⨁ High	MRAs result in reduced risk of hospitalization for heart failure.
**Worsening heart failure**	342 per 1,000	**281 per 1,000** (263 to 304)	**RR 0.82** (0.77 to 0.89)	6,467 (3 RCTs)	⨁⨁⨁⨁ High	MRAs reduce the risk of worsening heart failure.
**Quality of life** (assessed with MLHFQ) Follow-up: varying from 9 months to 12 months	Mean quality of life was 0	**MD 1.17 lower** (3.10 lower to 0.76 higher)	-	505 (3 RCTs)	⨁⨁◯◯ Low^b,c^	MRAs may not result in a difference in quality of life assessed by the MLHFQ.
**Quality of life** (assessed with KCCQ Overall Summary Score) Follow-up: varying from 6 months to 39.6 months	-	**MD 3.62 higher** (1.85 lower to 9.10 higher)	-	7,659 (3 RCTs)	⨁⨁◯◯ Low^b,d^	MRAs may not result in a difference in quality of life assessed by the KCCQ.
**Hyperkalemia** Follow-up: varying from 6 months to 39.6 months	59 per 1,000	**127 per 1,000** (111 to 128)	**RR 2.16** (1.89 to 2.47)	9,654 (5 RCTs)	⨁⨁⨁⨁ High	MRAs increase the risk of hyperkalemia.
**Worsening renal function** Follow-up: varying from 6 months to 32 months	18 per 1,000	**28 per 1,000** (17 to 44)	**RR 1.53** (0.96 to 2.45)	6,554 (3 RCTs)	⨁⨁⨁◯ Moderate^a,b^	MRAs probably result in worsening renal function.

CI: confidence interval; MRAs: mineralocorticoid receptor antagonists; RCTs: randomized clinical trials; RR: relative risk. The MLHFQ (Minnesota Living with Heart Failure Questionnaire) score ranges from 0 to 105, and higher scores indicate worse quality of life. The KCCQ (Kansas City Cardiomyopathy Questionnaire) score ranges from 0 to 100 in each domain, and higher scores indicate better quality of life. **Explanations:** a. Methodological limitations: Although some studies showed risk of bias, those contributing most to the analysis were classified as having low risk of bias. b. Imprecision: Wide confidence interval, indicating imprecision in the results. c. Methodological limitations: The RoB 2 tool classified the 2009 study by Mak et al.^22^ as having some concerns, with issues in the domains of randomization process and selective outcome reporting. The 2017 study by Upadhya et al.^23^ was also judged as having some concerns, with issues in the randomization process and deviations from intended interventions. d. Methodological limitations: The RoB 2 tool classified the 2011 RAAM-PEF24 and 2016 TOPCAT21 studies as having high risk of bias, with issues across multiple domains, compromising the reliability of the effect estimate. Source: the authors.

## Discussion

This systematic review evaluated the efficacy and safety of MRAs in patients with HFpEF and HFmrEF, demonstrating a consistent clinical benefit in terms of disease progression. Although the impact on mortality was limited, a statistically significant reduction in hospitalizations for heart failure was observed, indicating a relevant effect on patient morbidity. This benefit was present for both spironolactone and finerenone.

It is worth underscoring that, in the original publication of the TOPCAT study results, the analysis of the primary outcome component of hospitalizations for heart failure also demonstrated a benefit for spironolactone, with a hazard ratio of 0.83 (95% CI: 0.69 to 0.99; p = 0.04). Similarly, although the FINEARTS-HF study achieved a statistically significant reduction in the occurrence of the primary composite outcome of cardiovascular death or worsening heart failure events (first or recurrent unplanned hospitalization for heart failure or emergency room visit for heart failure), only the worsening heart failure component of the primary outcome showed a significant reduction with the use of finerenone (hazard ratio: 0.82; 95% CI: 0.71 to 0.94; p = 0.006), with a neutral impact on cardiovascular death (hazard ratio: 0.93; 95% CI: 0.78 to 1.11).

These results are similar to those of other studies testing treatments in patients with HFpEF or HFmrEF, showing benefits in reducing heart failure hospitalizations and events, yet without impact on overall or cardiovascular mortality, as observed in studies with SGLT2 inhibitors.^26,27^ Several analyses have suggested that the lack of impact on mortality outcomes in HFpEF or HFmrEF is due to the particular characteristics of this population, for example, advanced age and multiple comorbidities; as a result, the main cause of death is non-cardiovascular.^28^

In relation to quality of life, no statistically significant differences were identified between groups across the different scales assessed (MLHFQ, KCCQ, EQ5D-VAS, and SF-36). These results may suggest that MRAs have little impact on patients’ perceived well-being, even when other clinical parameters improve. This lack of effect may be attributed to variations in follow-up periods and differences in patients’ clinical characteristics among the included studies.

From a safety standpoint, therapy with MRAs has been associated with important adverse events, particularly a significant increase in the risk of hyperkalemia (with high certainty of evidence) and a trend toward worsening renal function (with moderate certainty of evidence). These findings highlight the need for careful individualized assessment of the risk-benefit ratio, especially in populations with a greater predisposition to electrolyte and renal disorders (for example, elderly patients and patients with chronic kidney disease). Likewise, the clinical management of these patients should include rigorous monitoring of serum creatinine and potassium levels.^29^

This systematic review followed a rigorous and transparent methodology, with broad and sensitive search strategies across multiple databases to identify relevant studies. Reviewers independently performed full-text selection and data extraction, reducing the risk of bias. The methodological quality of the RCTs was assessed using the RoB 2.0 tool, which allowed for detailed analysis of potential risks of bias in different domains, with outcome-based evaluation. Furthermore, the certainty of the evidence was classified according to the GRADE approach, reinforcing confidence in the findings and contributing to more informed clinical decision-making based on high-quality evidence.

The limitations of this study include a lack of standardization in the assessment of some outcomes, such as quality of life and worsening heart failure events, making direct comparison between studies difficult. Furthermore, as it is a rapid review, screening was performed by one reviewer and checked by a second reviewer, instead of independent double review, which may increase the risk of bias in the selection and extraction of data.

## Conclusion

The results of this meta-analysis support the recommendation to use MRAs, spironolactone or finerenone, in patients with HFpEF or HFmrEF to reduce hospitalizations and heart failure events (Central Illustration).

## Supplemental Materials

Material Suplementar
